# Impact of Evolocumab on LDL‐Cholesterol and Cardiovascular Outcomes in Patients With Established CVD: Systematic Review and Meta‐Analysis

**DOI:** 10.1002/edm2.70355

**Published:** 2026-10-08

**Authors:** Anika Goel, Muhammad Abdullah Naveed, Hemeesh Tandel, Affaf Mahmood, Muhammad Mukhlis, K. Sai Varun Reddy, Arun Kumar Maloth, Anchit Chauhan, Ameer Ashwara, Amogh Verma, Haris Bin Tahir

**Affiliations:** ^1^ Kakatiya Medical College Warangal Telangana India; ^2^ Dow Medical College Karachi Pakistan; ^3^ C U Shah Medical College Surendranagar Gujarat India; ^4^ Shaikh Khalifa Bin Zayed Al Nahyan Medical and Dental College Lahore Punjab Pakistan; ^5^ Ayub Medical College Abbottabad Pakistan; ^6^ Maulana Azad Medical College New Delhi India; ^7^ Department of Medicine An‐Najah National University Nablus Palestine; ^8^ SR Sanjeevani Hospital Siraha Nepal; ^9^ Dr. D. Y. Patil Medical College Hospital and Research Centre Dr. D. Y. Patil Vidyapeeth (Deemed‐to‐be‐University) Pune Maharashtra India; ^10^ Dr. D. Y. Patil Dental College and Hospital Dr. D. Y. Patil Vidyapeeth (Deemed‐to‐be‐University) Pune Maharashtra India; ^11^ Lahore General Hospital Lahore Pakistan

**Keywords:** evolocumab, LDL‐cholesterol, major adverse cardiovascular events, meta‐analysis, PCSK9 inhibitor, randomised controlled trials

## Abstract

**Background:**

Atherosclerotic cardiovascular disease is a leading cause of morbidity and mortality, and many high‐risk patients fail to reach LDL‐cholesterol (LDL‐C) targets on statins alone. Evolocumab, a PCSK9 inhibitor, offers potent LDL‐C lowering, but a synthesis of its lipid and clinical benefits in secondary prevention is needed.

**Methods:**

We conducted a systematic review and meta‐analysis of randomised controlled trials evaluating evolocumab in adults with established cardiovascular disease. PubMed, Embase, Scopus, Cochrane CENTRAL, and ClinicalTrials.gov were searched through April 2025. Outcomes included lipid parameters, major adverse cardiovascular events (MACE), individual cardiovascular endpoints, and safety, with pooled risk ratios (RR) and mean differences calculated using random‐effects models.

**Results:**

Nine trials enrolling 67,662 patients were included. Evolocumab significantly reduced LDL‐C (mean difference −57.73 mg/dL), total cholesterol (−52.27 mg/dL), triglycerides (−21.21 mg/dL), and lipoprotein(a) (−10.99 mg/dL), while increasing HDL‐cholesterol (3.69 mg/dL). Evolocumab was associated with lower MACE (RR 0.53; 95% CI 0.40–0.71), though this estimate should be interpreted cautiously given heterogeneity in endpoint definitions and study designs. Myocardial infarction (RR 0.74) and coronary revascularisation (RR 0.80) were significantly reduced, while cardiovascular mortality and stroke showed no statistically definitive reduction. Serious adverse events and neurocognitive effects were comparable between groups, though injection‐site reactions were more frequent with evolocumab.

**Conclusion:**

In patients with established cardiovascular disease, evolocumab provides substantial lipid lowering and reduces ischemic events without major safety concerns, supporting its use as an adjunct to statin therapy in secondary prevention.

## Graphical abstract

A systematic review and meta‐analysis of 9 randomised controlled trials (67,662 patients) found that evolocumab significantly improved lipid parameters and reduced major adverse cardiovascular events, myocardial infarction, and coronary revascularisation. No increase in serious adverse events or treatment discontinuation was observed, supporting its use in secondary cardiovascular prevention.

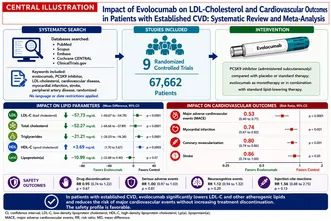

**Abbreviations**
ACC: American College of Cardiology
ACS: Acute Coronary Syndrome
AHA: American Heart Association
ASCVD: Atherosclerotic Cardiovascular Disease
CAD: Coronary Artery Disease
CENTRAL: Cochrane Central Register of Controlled Trials
CI: Confidence Interval
CVD: Cardiovascular Disease
EAS: European Atherosclerosis Society
ESC: European Society of Cardiology
EVOPACS: Evolocumab for Early Reduction of LDL Cholesterol in Acute Coronary Syndromes
FOURIER: Further Cardiovascular Outcomes Research With PCSK9 Inhibition in Subjects With Elevated Risk
FOURIER‐OLE: FOURIER Open‐Label Extension
HDL‐C: High‐Density Lipoprotein Cholesterol
LDL‐C: Low‐Density Lipoprotein Cholesterol
LDL‐R: Low‐Density Lipoprotein Receptor
Lp(a): Lipoprotein(a)
MACE: Major Adverse Cardiovascular Events
MD: Mean Difference
MI: Myocardial Infarction
PCSK9: Proprotein Convertase Subtilisin/Kexin Type 9
PRISMA: Preferred Reporting Items for Systematic Reviews and Meta‐Analyses
PROSPERO: International Prospective Register of Systematic Reviews
RCT: Randomised Controlled Trial
RevMan: Review Manager
RoB‐2: Risk of Bias 2 Tool
RR: Risk Ratio
SMD: Standardised Mean Difference
TC: Total Cholesterol
TG: Triglycerides

## 1 Introduction

Atherosclerotic cardiovascular disease (ASCVD) remains the leading global cause of morbidity and mortality, despite the widespread use of contemporary pharmacologic therapies [1]. Elevated low‐density lipoprotein cholesterol (LDL‐C) is a causal factor in ASCVD, and its reduction proportionally decreases cardiovascular risk. However, many high‐ and very‐high‐risk patients fail to achieve guideline‐recommended LDL‐C targets on statins alone, thereby carrying substantial residual risk [2, 3].

To address this gap, the 2018 AHA/ACC and 2019 ESC/EAS guidelines recommend adding non‐statin therapies such as ezetimibe or PCSK9 inhibitors for patients with ASCVD whose LDL‐C remains ≥ 70 mg/dL despite maximally tolerated statins [2, 4, 5]. Evolocumab, a fully human monoclonal antibody targeting PCSK9, enhances hepatic LDL receptor recycling and lowers LDL‐C by up to 60% [6].

Large randomised trials like FOURIER, FOURIER‐OLE, and EVOPACS have shown that evolocumab not only lowers LDL‐C to a profound extent but also reduces specific cardiovascular endpoints, particularly myocardial infarction and coronary revascularisation, with more limited effects observed for other individual outcomes [5, 7, 8]. Evolocumab also lowers lipoprotein(a) [Lp(a)]—a genetically driven, pro‐atherogenic particle not lowered by statins and increasingly appreciated as a contributor to residual cardiovascular risk [9]. Its effectiveness is also carried over to underrepresented populations like people with metabolic syndrome, without aggravating glycemia or diabetes occurrence [10].

Although the efficacy of evolocumab has been demonstrated in multiple trials, these studies differ in design, populations, follow‐up duration, and outcome measures. The FOURIER trial evaluated high‐risk ASCVD patients over 2.2 years [5], while FOURIER‐OLE extended follow‐up to nearly 8 years [6]. EVOPACS focused on acute coronary syndrome patients with short‐term follow‐up, and GLAGOV assessed plaque regression via intravascular ultrasound [7, 10]. A subgroup analysis also examined outcomes in patients with metabolic syndrome [3]. No prior meta‐analysis has comprehensively assessed evolocumab's effects on both lipid parameters and clinical outcomes in established ASCVD. This study aims to address that gap by synthesising data from randomised trials in secondary prevention populations.

Although previous systematic reviews and meta‐analyses have evaluated PCSK9‐targeted therapies as a therapeutic class, these analyses have generally pooled multiple agents—including evolocumab, alirocumab, and more recently inclisiran and other PCSK9‐targeted therapies—to estimate class‐wide treatment effects. For example, the Cochrane review by Schmidt et al. synthesised evidence from randomised trials of PCSK9 monoclonal antibodies for both primary and secondary prevention [11], while more recent network meta‐analyses by Zayed et al. [12], Raone et al. [13], and Mulligan et al. [14] included multiple PCSK9‐targeted agents across 8–31 randomised trials involving approximately 14,000–50,000 participants, evaluating outcomes ranging from cardiovascular events to LDL‐C and lipoprotein(a) reduction. Although these studies provide valuable class‐level evidence, combining different therapeutic agents introduces clinical heterogeneity arising from differences in clinical trial programs, patient populations, study designs, follow‐up durations, dosing regimens, and outcome reporting. Consequently, pooled estimates may not fully characterise the efficacy and safety profile of an individual agent. In contrast, the present study focuses exclusively on evolocumab, synthesising evidence from randomised controlled trials of a single PCSK9 inhibitor to provide drug‐specific estimates of lipid‐lowering efficacy, cardiovascular outcomes, and safety while minimising heterogeneity introduced by combining pharmacologically related but clinically distinct therapies.

Despite the availability of several randomised trials evaluating evolocumab, no previous meta‐analysis has comprehensively synthesised evidence exclusively from randomised controlled trials of evolocumab across both lipid and clinical cardiovascular outcomes in patients with established ASCVD. By focusing on a single PCSK9 inhibitor, the present study aims to provide a more clinically specific assessment of efficacy and safety while reducing heterogeneity associated with combining different agents within the PCSK9 inhibitor class.

## 2 Methods

This systematic review and meta‐analysis was conducted under PRISMA guidelines. The study protocol was prospectively registered in the International Prospective Register of Systematic Reviews (PROSPERO) under the registration number CRD420251043680. We searched PubMed, Scopus, Embase, Cochrane CENTRAL, and Clinical trials from inception through April 2025 for studies (randomised controlled trials) evaluating the efficacy and safety of evolocumab in patients with established cardiovascular disease (CVD). Search terms included ‘evolocumab’, ‘PCSK9 inhibitor’, ‘LDL‐cholesterol’, ‘cardiovascular disease’, ‘myocardial infarction’, ‘stroke’, ‘peripheral artery disease’, and ‘randomised’, adapted for each database. We also screened reference lists of relevant articles, clinical trial registries, and conference proceedings. A detailed search strategy is presented in (Table S1).

### 2.1 Inclusion and Exclusion Criteria

We included studies involving adult patients (≥ 18 years) with established CVD—defined as prior myocardial infarction, stroke, symptomatic peripheral artery disease, or coronary revascularisation—who received evolocumab either as monotherapy or in combination with standard lipid‐lowering therapy. Only randomised controlled trials comparing evolocumab to placebo or standard therapy were eligible. Trials were required to report at least one pre‐specified efficacy or safety outcome. We excluded non‐comparative studies, trials involving PCSK9 inhibitors other than evolocumab, mixed‐population studies lacking separable data for the CVD subgroup, and trials involving paediatric or special populations.

### 2.2 Study Selection and Data Extraction

Two reviewers independently extracted data from eligible studies using a standardised data extraction form developed specifically for this review. The extraction form was pilot‐tested on a subset of included studies and refined to ensure consistency and completeness of data collection. Extracted information included study characteristics, baseline patient demographics, clinical presentation, intervention details, comparator characteristics, follow‐up duration, and efficacy and safety outcomes. Any discrepancies between reviewers during data extraction were resolved through discussion and consensus; if agreement could not be reached, a third reviewer was consulted for final adjudication. Missing data were handled by extracting all available information from the primary publications, Supporting Information, and related trial reports. When outcome data were unavailable or insufficiently reported, these outcomes were not imputed and were analysed only when extractable data were available. Multiple publications arising from the same randomised trial were identified by comparing study characteristics, recruitment periods, sample sizes, baseline demographics, and intervention details. To avoid double‐counting of participants, data from the most comprehensive or most recent publication reporting the relevant outcome were preferentially included. The detailed PRISMA Flowchart comprehensively explaining the study selection process is illustrated in Figure 1. Outcome data were extracted from intention‐to‐treat populations as reported in the original randomised controlled trials.

**FIGURE 1:**
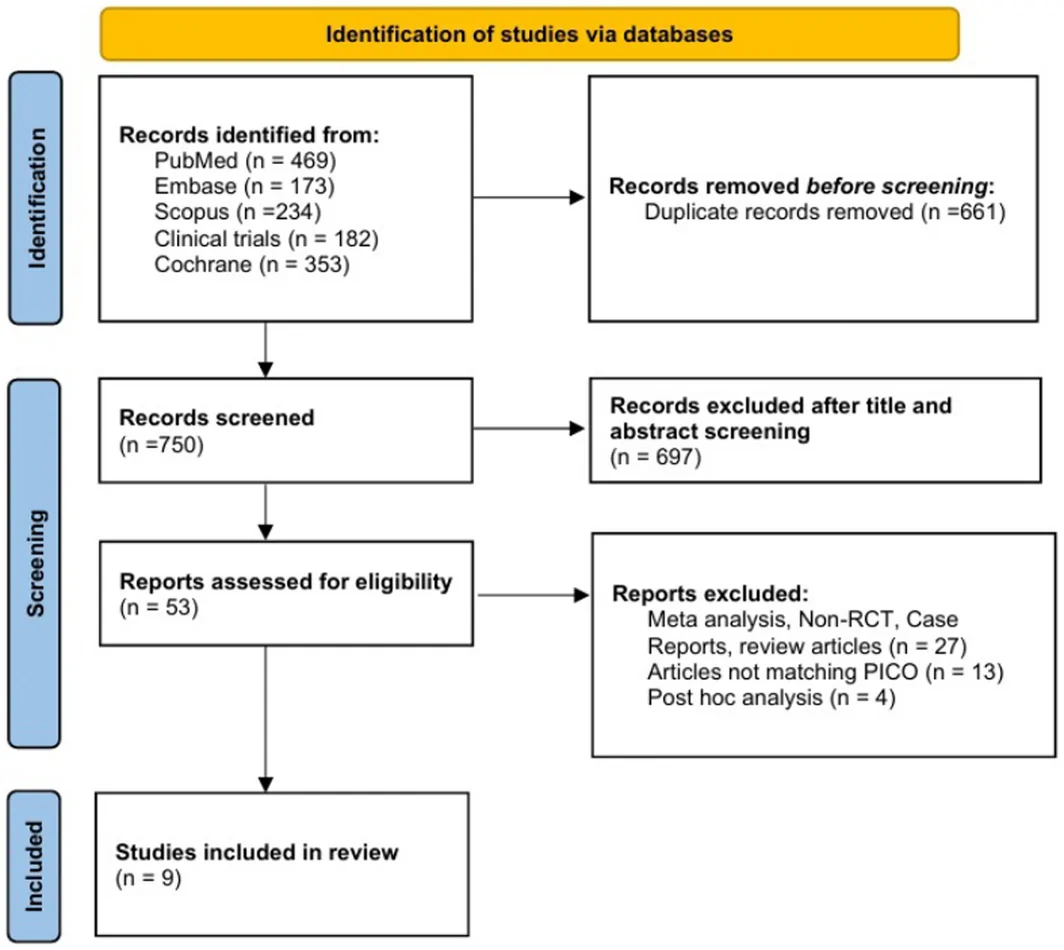
PRISMA flow diagram.

### 2.3 Primary and Secondary Outcomes

The primary outcomes included absolute changes in lipid parameters (total cholesterol, LDL‐cholesterol, HDL‐cholesterol, triglycerides, and Lp(a)) and incidence of major adverse cardiovascular events (MACE). MACE was analysed according to the composite endpoint definition reported in each individual trial, which generally included myocardial infarction, stroke, and cardiovascular death; however, minor variations in component definitions across studies were considered during interpretation. Secondary outcomes included individual rates of MI, stroke, coronary revascularisation, and all‐cause mortality. Additional events like injection‐site reactions, musculoskeletal and neurocognitive side effects were also assessed.

#### 2.3.1 Risk of Bias Assessment

The risk of bias for all randomised controlled trials was assessed using the Cochrane Risk of Bias 2 (RoB‐2) tool (Figure 2). Two reviewers independently rated each domain, and discrepancies were resolved by consensus.

**FIGURE 2:**
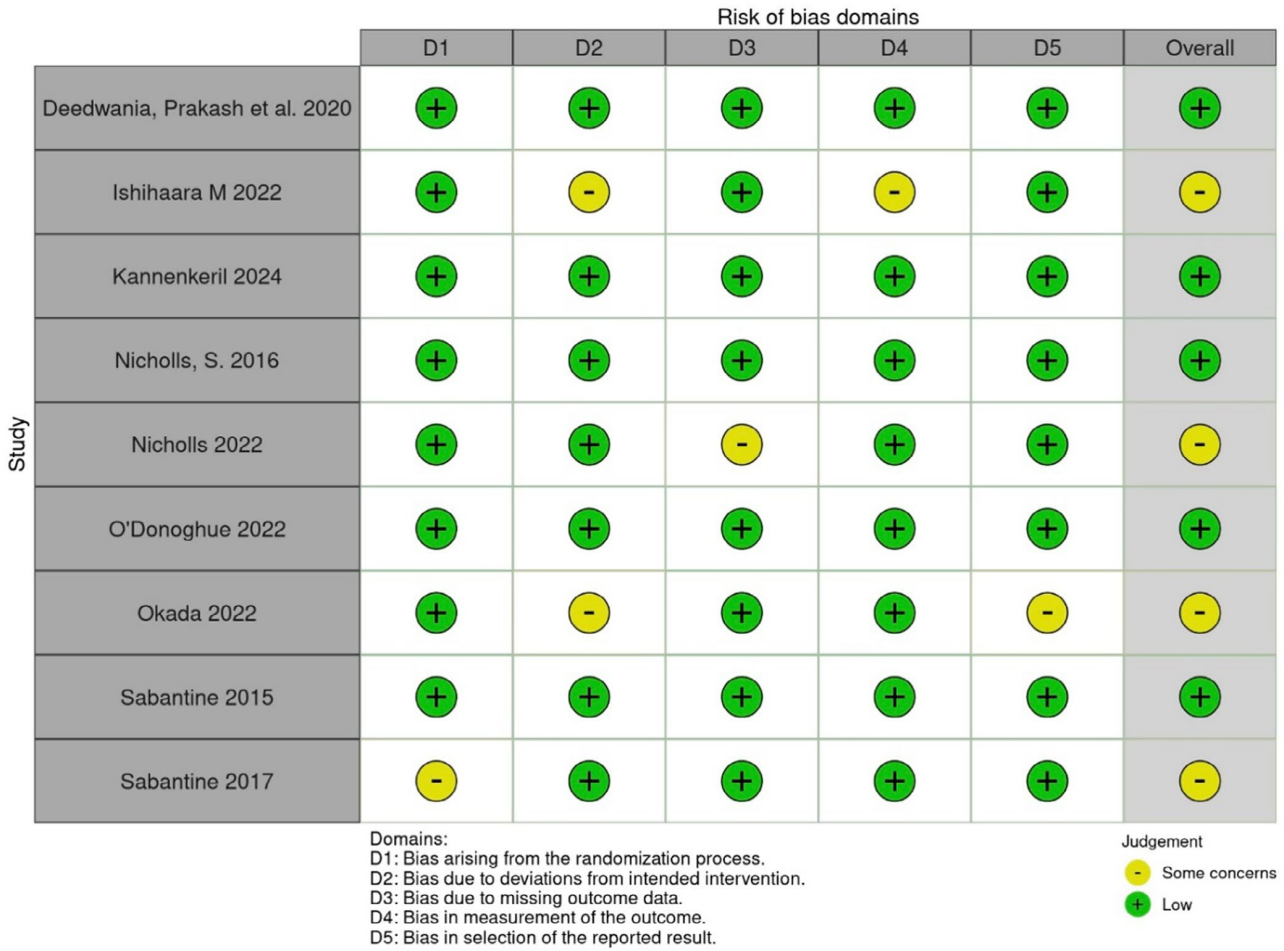
Risk of bias (RoB) assessment.

### 2.4 Statistical Analysis

Pooled analyses were performed using a random‐effects model. For dichotomous outcomes, we calculated pooled risk ratios (RR) with 95% confidence intervals (CI). For continuous outcomes, we used mean differences (MD) or standardised mean differences (SMD) as appropriate. Statistical heterogeneity was assessed using the *I* ^2^ statistic, with values > 50% considered substantial. Analyses were conducted using Review Manager (RevMan 5.4), and *p*‐values < 0.05 were considered statistically significant.

In addition, predefined subgroup analyses were conducted to explore potential sources of heterogeneity and assess the consistency of treatment effects across clinically and methodologically relevant strata. These analyses were conducted not only to assess statistical robustness but also to explore whether differences in clinical presentation, trial objectives, and follow‐up duration contributed to variability in treatment effects. Subgroups were stratified according to: (1) clinical presentation, comparing acute coronary syndrome (ACS) versus stable coronary artery disease/atherosclerotic cardiovascular disease (stable CAD/ASCVD), where ACS refers to trials enrolling patients with acute myocardial ischemic syndromes and stable CAD/ASCVD refers to chronic, non‐acute atherosclerotic disease populations; (2) trial design, categorised as event‐driven trials (studies primarily designed to evaluate cardiovascular clinical outcomes), lipid‐only trials (studies primarily assessing lipid parameter changes without clinical endpoints), mechanistic trials (studies evaluating biological or physiological effects of Evolocumab), and imaging/atherosclerosis structure trials (studies assessing plaque burden, vascular morphology, or atherosclerotic progression using imaging modalities); and (3) duration of follow‐up, categorised as short‐term (< 1 year) and long‐term (≥ 1 year) follow‐up studies (Figures S23–S45).

Trials where time‐to‐event outcomes were reported as hazard ratios in individual trials, these were not pooled directly with risk ratios. Instead, where available, event data were extracted and synthesised using risk ratios (RR) to maintain consistency of effect measures across studies. No conversion of hazard ratios to risk ratios was performed. The certainty of evidence for all efficacy and safety outcomes was assessed using the Grading of Recommendations Assessment, Development and Evaluation (GRADE) framework, considering risk of bias, inconsistency, indirectness, imprecision, and publication bias (Table S2).

## 3 Results

### 3.1 Study Characteristics

A total of 67,662 patients were included in this meta‐analysis, with 34,618 receiving Evolocumab (intervention group) and 33,044 receiving standard therapy (control group). Every study was multicentre and conducted across multiple international sites. The mean age of participants ranged from 57.8 to 68.1 years for male participants. Comprehensive baseline characteristics are mentioned in Table 1.

**TABLE 1:** Baseline characteristics of included studies.

Study ID	Country	Study design	Prospective or retrospective, duration	Sample size (Intervention vs. Control)	Age, years (Mean, SD)		Male *n* (%)		Hypertension *n* (%)		Diabetes *n* (%)		Current smoking *n* (%)	
					Intervention	Control	Intervention	Control	Intervention	Control	Intervention	Control	Intervention	Control
Huang et al., 2021	China	Multicenter RCT	Prospective, 2019 to 2020	64 (32 vs. 32)	58.4 (14.6)	57.4 (13.6)	16 (50.0)	14 (43.8%)	17 (53.1%)	16 (50.0%)	N/A	N/A	4 (12.5%)	3 (9.4%)
Ishihara et al., 2022	Japan	Multicenter RCT	Prospective, 2019 to 2021	100 (54 vs. 46)	68.1 (10.3)	67.0 (11.1)	40 (74)	35 (76%)	40 (74%)	37 (80%)	20 (37%)	20 (43%)	10 (19%)	12 (26%)
Kannenkeril et al., 2024	Germany	Single center RCT	Prospective, 2019 to 2021	103 (53 vs. 50)	66.9 (6.9)	65.5 (8.5)	86.80%	92%	81.10%	76.00%	17%	18%	N/A	N/A
O'Donoghue et al., 2022	USA, Europe	Multicenter Extensional RCT	Prospective, 2016 to 2017	6635 (3355 vs. 3280)	62.4 (8.6)	62.4 (8.6)	2582 (77.0)	2504 (76.3)	82.50%	84.50%	33.40%	34.90%	26.30%	27.10%
Nicholls et al., 2016	North America, Europe, South America, Asia, Australia, and South Africa	Multicenter RCT	Prospective, 2013 to 2015	968 (484 vs. 484)	59.8 (9.6)	59.8 (8.8)	349 (72.1)	350 (72.3)	398 (82.2)	405 (83.7)	98 (20.2)	104 (21.5)	124 (25.6)	113 (23.3)
Nicholls et al., 2022	Japan, USA, Australia, Netherlands, Hungary, Italy	Multicenter RCT	Prospective, 2018 to 2019	161 (80 vs. 81)	60.9 10.0	60.2 9.2	60 (75.0)	55 (67.9)	45 (56.3)	33 (40.7)	13 (16.3)	14 (17.3)	47 (58.8)	48 (59.3)
Okada et al., 2022	Japan	Single center Sub‐analysis of RCT	Prospective, 2017 to 2019	102 (52 vs. 50)	66.4 (13.1)	63.4 (14.0)	43 (82)	47 (94)	31 (59)	33 (66)	17 (32)	14 (28)	25 (48)	18 (36)
Sabatine et al., 2015	United states	Multicenter Exploratory of OSLER‐1 and OSLER‐2 RCT	Prospective, 2011 to 2014	4465 (2976 vs. 1489)	57.8 ± 11.0	58.2 ± 10.9	1490 (50.1)	765 (51.4)	1545 (51.9)	777 (52.2)	382 (12.8)	217 (14.6)	465 (15.6)	222 (14.9)
Sabatine et al. 2017	United states, Australia, United Kingdom, Norway	Multicenter RCT	Prospective, 2013 to 2015	27,564 (13,784 vs. 13,780)	62.5 ± 9.1	62.5 ± 8.9	10,397 (75.4)	10,398 (75.5)	80.10%	80.10%	5054 (36.7)	5027 (36.5)	28%	28.50%
Deedwania et al., 2020	United states, United Kingdom, Australia, California, Norway, Belgium, Lithuania	Post hoc analysis of RCT	Prospective, 2013 to 2015	27,564 (13,780 vs. 13,784)	63.3 (9.1)		8883 (80.9)		7521 (68.5)		N/A	N/A	3319 (30.2)	

Abbreviations: %, percentage; *n*, number of participants; N/A, not available; RCT, randomised controlled trial; SD, standard deviation; USA, United States of America.

### 3.2 Primary Outcomes

#### 3.2.1 Outcome Measures of Effectiveness

This meta‐analysis systematically evaluated the effects of Evolocumab on changes in lipid profile and MACE in the population. Outcomes and safety measures are also revealed in this study. The analysis included key biomarkers of the Lipid Profile such as total cholesterol (TC), triglycerides (TG), high‐density lipoprotein cholesterol (HDL‐C), Low Density Lipoprotein (LDL) and Lp(a) levels.

#### 3.2.2 Total Cholesterol

The pooled analysis of TC has significant heterogeneity (*p* < 0.001, *I* ^2^ = 100%) (Figure 3). However, the random‐effects model revealed that Evolocumab significantly reduced TC levels compared to placebo (MD = −52.27 mg/dL, 95% CI −66.66 to −37.89 mg/dL), highlighting the potential of decreasing the levels of TC in the population.

**FIGURE 3:**
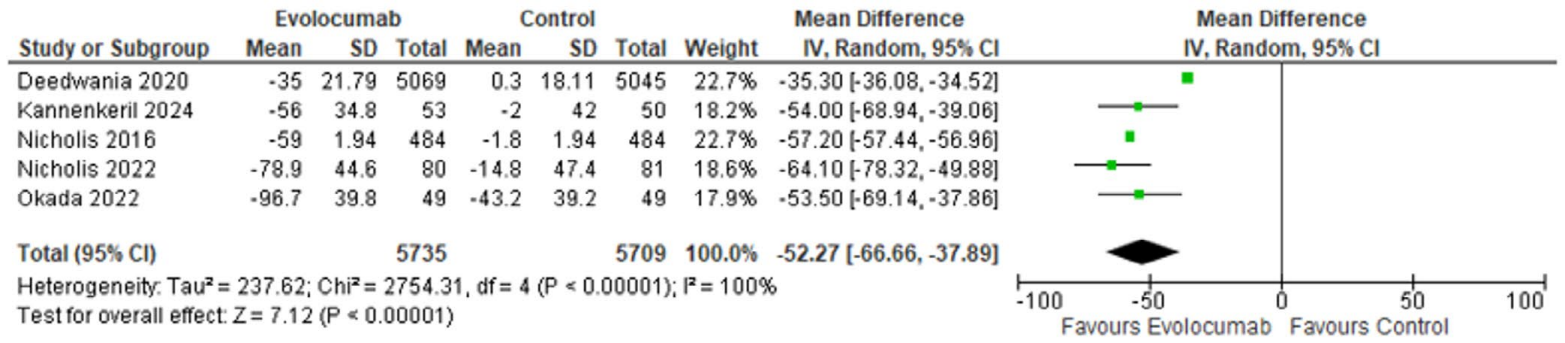
Forest plot showing changes in total cholesterol level.

#### 3.2.3 Triglycerides

The pooled analysis of TG, having 6 studies and 11,526 participants (5779 in the treatment group and 5747 in the control group) indicated that Evolocumab significantly reduced TG levels (MD = −21.21 mg/dL, 95% CI −26.03 to −16.38 mg/dL) amid significant heterogeneity (*p* < 0.001, *I* ^2^ = 92%) (Figure 4).

**FIGURE 4:**
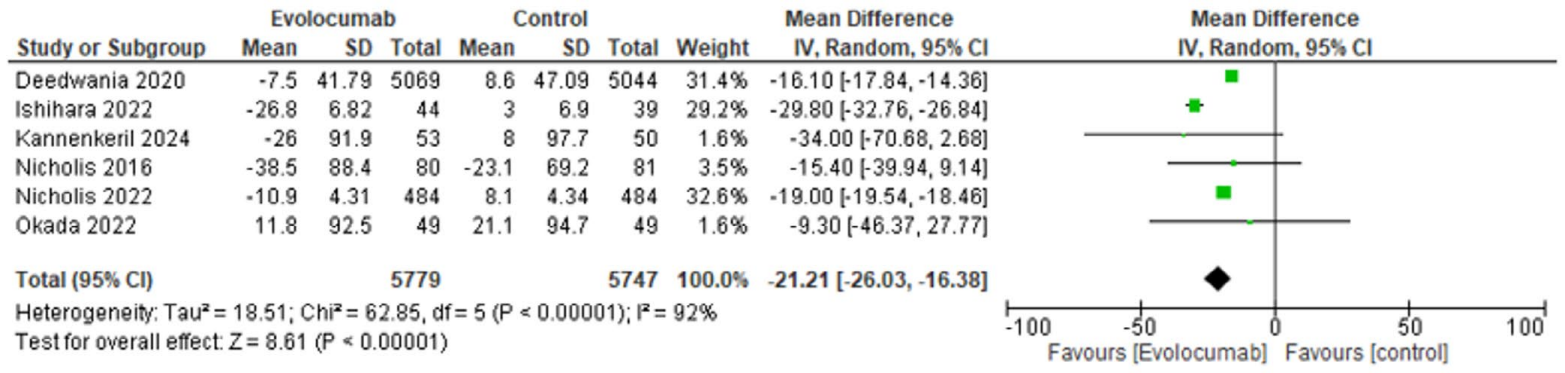
Forest plot showing changes in triglyceride level.

#### 3.2.4 HDL

The pooled analysis of HDL, covering 6 studies with 11,536 participants (5788 in the treatment group and 5748 in the control group), revealed that Evolocumab significantly increased HDL levels (MD = 3.69 mg/dL, 95% CI 1.70–5.67 mg/dL) despite significant heterogeneity (*p* < 0.001, *I* ^2^ = 98%) highlighting the potential of increasing the HDL levels in the population (Figure 5).

**FIGURE 5:**
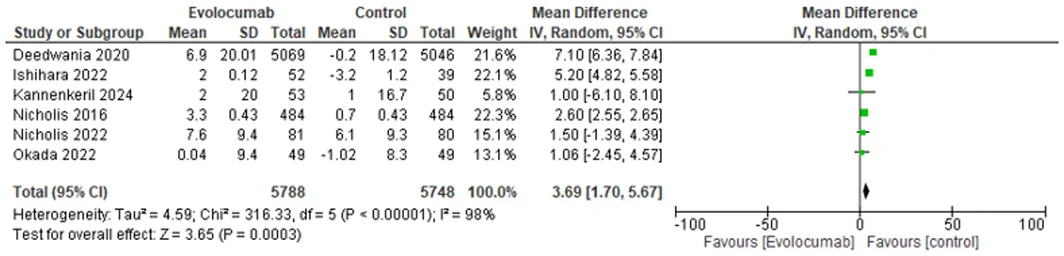
Forest plot showing changes in High Density Lipoprotein (HDL) levels.

#### 3.2.5 LDL

The pooled analysis of LDL, covering 5 studies with 4008 participants in the treatment group and 3950 participants in the control group, revealed a statistically significant reduction of LDL in the treatment group compared to the control group due to the effect of Evolocumab (MD = −57.73 mg/dL; 95% CI −60.67 to −54.78, *p* < 0.001). This analysis had substantial heterogeneity (*I* ^2^ = 91%) (Figure 6).

**FIGURE 6:**
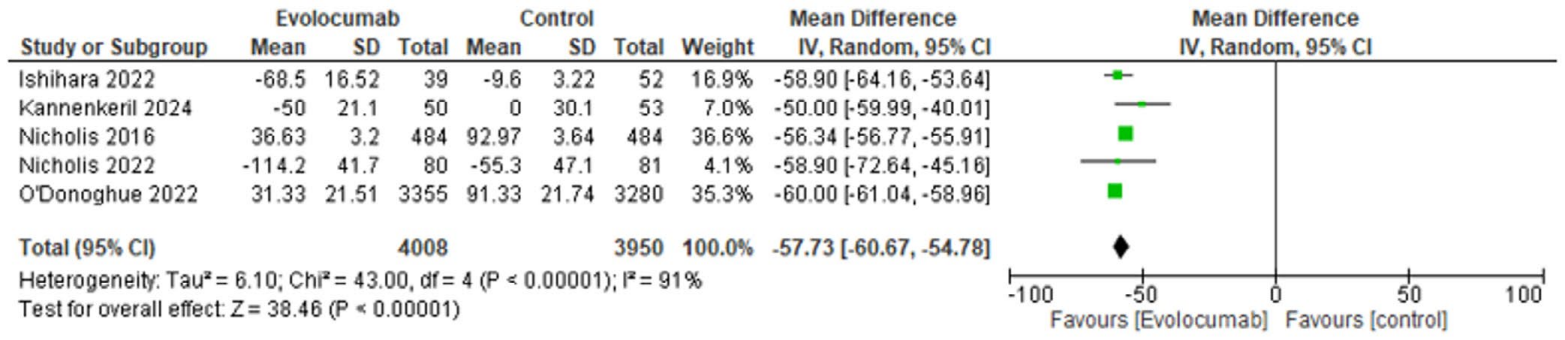
Forest plot showing changes in Low Density Lipoprotein (LDL) levels.

#### 3.2.6 LP(a)

For Lp(a), the pooled analysis of 5 studies with 10,720 participants (5360 in the treatment group and 5360 in the control group) showed a reduction in Lp(a) levels with Evolocumab compared with controls (MD = −10.99 mg/dL, 95% CI −22.89 to 0.90 mg/dL); however, this reduction did not reach statistical significance (*p* = 0.07) (Figure S1).

Despite statistically significant pooled effects for most lipid parameters, substantial between‐study heterogeneity was observed for several outcomes. Therefore, these estimates should be interpreted as average treatment effects across diverse trial populations rather than as a uniform magnitude of lipid reduction expected in all clinical settings. Differences in baseline lipid levels, background lipid‐lowering therapy, study objectives, and follow‐up duration may have contributed to variability in observed treatment effects.

### 3.3 Secondary Outcomes

#### 3.3.1 MACE

The pooled analysis of major adverse cardiovascular events (MACE), defined according to individual trial composite endpoint definitions, included 5 studies with 39,793 participants, 20,679 participants in the treatment group and 19,114 participants in the control group. All included trials were harmonised using dichotomous event data, and pooled effect estimates were calculated as risk ratios (RR) rather than hazard ratios (HR). There was substantial heterogeneity (*I* ^2^ = 95%) present in this analysis. Although the random‐effects model demonstrated a statistically significant reduction in MACE (RR = 0.53; 95% CI 0.40–0.71; *I* ^2^ = 95%), the high degree of between‐study variability indicates that the magnitude of the observed treatment effect should be interpreted cautiously and may not represent a uniform reduction across all clinical populations (Figure S2).

Further analysis of several key outcomes was conducted which showed that there was no statistically significant reduction in the outcomes like cardiovascular deaths (RR = 0.92; 95% CI 0.79–1.07; *p* = 0.30, *I* ^2^ = 48%) (Figure S3) and Hospitalisation in Unstable Angina (RR = 0.98; 95% CI 0.82–1.16, *p* = 0.78, *I* ^2^ = 0%) (Figure S4). However there was a statistically significant reduction seen in the events of outcomes like Stroke (RR = 0.86; 95% CI 0.74–1.00; *p* = 0.05, *I* ^2^ = 5%) (Figure S5), Myocardial Infarction (MI) (RR = 0.74; 95% CI of 0.67–0.82, *p* < 0.001, *I* ^2^ = 0%) (Figure S6), and Coronary Revascularisation (RR = 0.80; 95% CI 0.74–0.86, *p* < 0.001, *I* ^2^ = 31%) (Figure S7).

### 3.4 Outcome Indicators for Safety

#### 3.4.1 Serious Adverse Events

To evaluate the safety of this PCSK9 drug Evolocumab in the patients, we comprehensively analysed the incidence of serious adverse events, serious adverse events leading to drug discontinuation and other adverse events. Six studies including 28,845 participants in the treatment group and 27,246 participants in the control group, with no significant heterogeneity (*I* ^2^ = 0%), which conveys consistent results, revealed that there is no statistically significant difference in the incidence of serious adverse events in the treatment group as compared to the control group (RR = 1; 95% CI 0.97–1.03; *p* = 0.81) (Figure S8).

#### 3.4.2 Drug Discontinuation

Further meta‐analysis of serious adverse events leading to drug discontinuation involving 3 studies with 17,202 participants in the treatment group and 17,114 participants in the control group showed substantial heterogeneity of (*I* ^2^ = 70%). There is no statistically significant difference in the events of drug discontinuation in the treatment group compared to the control group (RR = 0.95; 95% CI 0.74–1.22; *p* = 0.67) (Figure S9).

#### 3.4.3 Other Adverse Events

Regarding other adverse events, we have comprehensively analysed three different types of adverse events like Neurocognitive adverse events, Injection site reactions events and muscle‐related adverse events. The pooled analysis of Neurocognitive adverse events shows there is not a significant statistical reduction in the events in the treatment group as compared to the control group (RR = 1.12; 95% CI 0.94–1.32; *p* = 0.20, *I* ^2^ = 33%) (Figure S10). Pooled analysis of Incidence of Injection site reaction also did not show significant statistical reduction of events in the treatment group as compared to the control group (RR = 1.56; 95% CI 0.88–2.75; *p* = 0.13, *I* ^2^ = 67%) (Figure S11), and lastly we have compared Muscle related adverse events in the treatment group with the control groups which also showed no statistical significant difference (*R* = 1.05; 0.97 to 1.15, *p* = 0.24, *I* ^2^ = 0%) (Figure S12). This comprehensive analysis highlights the safety profile of PCSK9 inhibitor Evolocumab, particularly concerning serious adverse events, serious adverse reactions leading to drug discontinuation and also showed details of safety information for its clinical use like Local injection site reaction, Neurocognitive adverse events and Muscle related adverse events.

### 3.5 Sensitivity Analysis

The sensitivity analysis demonstrated substantial heterogeneity across several outcomes with *I* ^2^ values greater than 50%. To evaluate the robustness and stability of the pooled estimates, a leave‐one‐out sensitivity analysis was performed by sequentially excluding one study at a time to determine its influence on heterogeneity and overall significance. For Total Cholesterol (5 studies), heterogeneity was initially extremely high (*I* ^2^ = 100%, *p* < 0.00001). After exclusion of Deedwania et al., heterogeneity was completely resolved (*I* ^2^ = 0%) while the pooled effect remained statistically significant (*p* < 0.00001), indicating that this study was the primary contributor to inconsistency. For Triglycerides (6 studies), initial heterogeneity was considerable (*I* ^2^ = 96%, *p* < 0.00001). Removal of Ishihara et al. reduced heterogeneity to 63%, while the pooled effect remained highly significant (*p* < 0.00001), suggesting partial contribution of this study to the observed heterogeneity. Similarly, for HDL (6 studies), heterogeneity was initially very high (*I* ^2^ = 99%, *p* = 0.0009). Excluding Nichols et al. reduced heterogeneity to 89%, with the pooled analysis still demonstrating statistical significance (*p* < 0.00001), indicating persistent residual heterogeneity despite study removal. For LDL (5 studies), substantial heterogeneity was observed initially (*I* ^2^ = 88%, *p* < 0.00001). After excluding O'Donoghue et al., heterogeneity decreased to 0% while maintaining strong statistical significance (*p* < 0.00001), demonstrating that this study largely accounted for heterogeneity. For Lp(a) (5 studies), heterogeneity remained high (*I* ^2^ = 99%, *p* = 0.06). Exclusion of Nichols et al. slightly reduced heterogeneity to 98%, while the pooled effect became statistically significant (*p* = 0.01), suggesting persistent between‐study variability. For drug discontinuation (3 studies), heterogeneity was initially moderate (*I* ^2^ = 67%, *p* = 0.67). Following exclusion of Sabatine et al., heterogeneity decreased to 46%, while the pooled estimate remained non‐significant (*p* = 0.35). Finally, for injection site reactions (5 studies), heterogeneity was markedly high (*I* ^2^ = 97%, *p* = 0.24). After removal of Sabatine et al., heterogeneity was completely resolved (*I* ^2^ = 0%) and the pooled effect became statistically significant (*p* = 0.0004), indicating that this study substantially influenced the inconsistency across studies (Table S3) (Figures S13–S19).

Despite sensitivity analysis, substantial residual heterogeneity persisted in outcomes including Triglycerides, HDL, and Lp(a) with *I* ^2^ values remaining greater than 50% after exclusion of influential studies. Therefore, the pooled estimates for these outcomes should be interpreted cautiously due to the presence of considerable between‐study variability.

#### 3.5.1 Sensitivity Analysis (MACE)

To assess the robustness of the pooled estimate and explore the source of heterogeneity, sensitivity analyses were performed by sequentially excluding studies with disproportionate influence on effect size and heterogeneity (Table S3).

##### 3.5.1.1 Exclusion of Sabatine 2015 Study

After removal of the Sabatine 2015 trial, heterogeneity decreased slightly from 95% to 88%, while the overall effect remained statistically significant (*p* = 0.0006), indicating that this study contributed modestly to between‐study variability but did not alter the direction of effect (Figure S20).

##### 3.5.1.2 Exclusion of Two Small Studies (Nicholls 2016 and Nicholls 2022)

Given the concern for small‐study effects and potentially exaggerated effect sizes, both Nicholls 2016 and Nicholls 2022 were excluded. This resulted in an increase in heterogeneity (*I* ^2^ = 97%), suggesting that these studies were not the primary drivers of heterogeneity and that variability was influenced by other between‐study differences (Figure S21).

##### 3.5.1.3 Exclusion of Sabatine 2015 Along With Both Small Studies (Nicholls 2016 and Nicholls 2022)

When both small studies and the Sabatine 2015 trial were simultaneously excluded, heterogeneity was completely eliminated (*I* ^2^ = 0%), while the pooled effect remained statistically significant and consistent in magnitude (RR = 0.84, 95% CI: 0.81–0.88; *p* < 0.001) (Figure S22).

The sensitivity analyses demonstrate that although substantial heterogeneity (*I* ^2^ = 95%) was present in the primary analysis, this heterogeneity is largely driven by a small subset of studies with differing effect sizes and sample characteristics. Importantly, exclusion of these studies does not materially alter the overall direction or statistical significance of the effect. Across all sensitivity scenarios, Evolocumab consistently demonstrates a reduction in MACE. Therefore, the pooled estimate is robust, and the observed heterogeneity is attributable to study‐level variability rather than inconsistency in the treatment effect.

### 3.6 Subgroup Analyses

Subgroup analyses were conducted according to clinical presentation, trial design, and follow‐up duration for lipid outcomes, MACE, treatment discontinuation, and injection‐site reactions (Figures S23–S45). For total cholesterol, significant reductions were observed in both ACS (MD = −59.31 mg/dL, 95% CI −69.82 to −48.79; *I* ^2^ = 0%) and stable CAD/ASCVD populations (MD = −48.48 mg/dL, 95% CI −66.57 to −30.38; *I* ^2^ = 100%) (Figures S23–S25). Similarly, LDL‐C reductions remained significant across all subgroup categories, with effect estimates ranging from −55.51 to −60.00 mg/dL. No heterogeneity was observed among imaging trials (*I* ^2^ = 0%) (Figures S26–S28). Triglyceride reductions were significant across most subgroup categories, although no significant effect was observed in the lipid‐only trial subgroup (MD = −9.30 mg/dL, 95% CI −46.37 to 27.77; *p* = 0.62) (Figures S29–S31).

For HDL‐C, significant increases were observed in stable CAD/ASCVD populations (MD = 4.62 mg/dL, 95% CI 2.26–6.99; *p* = 0.0001), event‐driven trials (MD = 7.10 mg/dL, 95% CI 6.36–7.84; *p* < 0.001), mechanistic trials (MD = 4.66 mg/dL, 95% CI 1.90–7.42; *p* = 0.0009), and studies with follow‐up duration ≥ 1 year (MD = 3.87 mg/dL, 95% CI 0.27–7.46; *p* = 0.03), whereas no significant effect was observed in ACS populations or lipid‐only trials (Figures S32–S34). For Lp(a), significant reductions were observed in ACS populations (MD = −12.45 mg/dL, 95% CI: −23.61 to −1.29; *p* = 0.03), event‐driven trials (MD = −27.90 mg/dL, 95% CI −30.61 to −25.19; *p* < 0.001), and studies with follow‐up duration < 1 year (MD = −6.57 mg/dL, 95% CI −8.70 to −4.44; *p* < 0.001), whereas no significant reductions were observed among stable CAD/ASCVD populations, imaging trials, or studies with follow‐up duration ≥ 1 year (Figures S35–S37).

For MACE, a significant reduction was observed among stable CAD/ASCVD studies (RR = 0.54, 95% CI 0.40–0.72; *p* < 0.0001), imaging trials (RR = 0.46, 95% CI 0.36–0.59; *p* < 0.00001), event‐driven trials (RR = 0.58, 95% CI 0.42–0.78; *p* = 0.0004), and studies with follow‐up duration ≥ 1 year (RR = 0.73, 95% CI 0.61–0.87; *p* = 0.0006). No statistically significant reduction was observed in the ACS subgroup (RR = 0.11, 95% CI 0.01–2.06; *p* = 0.14) (Figures S38–S40). No significant differences in treatment discontinuation were observed across any subgroup category (Figures S41 and S42). For injection‐site reactions, no significant differences were observed across clinical presentation or trial‐design subgroups. Significant increases were observed in studies with follow‐up duration < 1 year (RR = 129.63, 95% CI 8.07–2082.13; *p* = 0.0006) and ≥ 1 year (RR = 1.33, 95% CI 1.14–1.55; *p* = 0.0004) (Figures S43–S45). Detailed subgroup effect estimates and forest plots are provided in the Supporting Information (Figures S23–S45).

### 3.7 Certainty of Evidence Assessment

The certainty of evidence for all assessed outcomes is summarised in Table S2. Evidence quality was rated as high for myocardial infarction, coronary revascularisation, stroke, and serious adverse events. Moderate‐certainty evidence was observed for total cholesterol, triglycerides, HDL‐C, LDL‐C, major adverse cardiovascular events, cardiovascular mortality, drug discontinuation due to adverse events, neurocognitive adverse events, and injection‐site reactions, primarily due to heterogeneity and/or imprecision. The certainty of evidence for lipoprotein(a) was rated as low because of substantial heterogeneity and confidence intervals crossing the line of no effect.

### 3.8 Publication Bias

Egger's regression test was not applied, consistent with methodological consensus that it is unsuitable for meta‐analyses with fewer than 10 included studies.

## 4 Discussion

In this large meta‐analysis of nine randomised trials (67,662 patients) [3, 5, 7, 15, 16, 17, 18, 19, 20], evolocumab was associated with profound lipid lowering and a significant reduction in ischemic cardiovascular events in patients with established CVD. Our pooled estimates showed that evolocumab on a statin background lowered LDL‐cholesterol by roughly 50%–60% and reduced total cholesterol and triglycerides, modestly raised HDL‐cholesterol, and markedly lowered Lp(a). Importantly, statistical significance of individual lipid changes does not necessarily indicate equivalent clinical importance. Among these parameters, LDL‐C reduction represents the principal established mediator of cardiovascular risk reduction with PCSK9 inhibition, given the causal relationship between cumulative LDL‐C exposure and atherosclerotic cardiovascular disease. In contrast, the modest increases in HDL‐C and reductions in triglycerides observed in our analysis are unlikely to independently account for a substantial proportion of the cardiovascular benefit associated with evolocumab and should be interpreted primarily as accompanying metabolic effects rather than major drivers of event reduction. Importantly, the clinical interpretation of lipid changes should consider treatment targets rather than population‐based physiological reference ranges alone. Although evolocumab produced substantial reductions in LDL‐C, the achieved LDL‐C concentrations may not uniformly correspond to the conventional desirable range observed in the general population (< 100 mg/dL). However, in patients with established ASCVD, contemporary prevention strategies prioritise achieving individualised guideline‐based LDL‐C targets, with very‐high‐risk patients recommended to achieve LDL‐C levels below 55–70 mg/dL depending on guideline framework and clinical risk. Therefore, the clinical relevance of evolocumab therapy lies not only in normalisation of lipid parameters but in achieving intensive LDL‐C lowering associated with proportional reductions in cardiovascular risk. Similarly, although triglyceride levels demonstrated statistically significant reductions, the magnitude of change was modest and may not consistently result in normalisation below the commonly accepted threshold of 150 mg/dL. HDL‐C increases were also modest and may not necessarily translate into additional cardiovascular protection, as pharmacologically mediated HDL‐C elevation has not consistently demonstrated outcome benefits. Collectively, these findings support the interpretation that LDL‐C reduction remains the predominant therapeutic mechanism underlying the cardiovascular benefit observed with evolocumab, whereas changes in triglycerides and HDL‐C represent secondary metabolic effects. These metabolic effects were associated with a reduction in major adverse cardiovascular events (MACE), including myocardial infarction and coronary revascularisation, although the magnitude of the pooled treatment estimate should be interpreted cautiously given substantial heterogeneity across included studies and methodological differences between trials. Importantly, the pooled MACE estimate in this meta‐analysis should not be interpreted as indicating that evolocumab uniformly reduces cardiovascular events by approximately 50%. The observed effect size is larger than that reported in major event‐driven trials such as FOURIER and likely reflects differences in statistical methodology, including the pooling of risk ratios from aggregated event counts rather than hazard ratios from time‐to‐event analyses, as well as variation in study populations, endpoint definitions, follow‐up duration, and inclusion of smaller trials with fewer cardiovascular events. Given the substantial heterogeneity observed across several pooled outcomes, particularly lipid parameters and MACE, the magnitude of these effects should be interpreted with appropriate caution. These findings mirror and extend results from the FOURIER and EVOPACS trials, which found similar LDL‐C reductions (~59%–60%) and event reductions when evolocumab was added to high‐intensity statin therapy [5, 8]. Mechanistically, this is expected, since evolocumab is a human monoclonal antibody to PCSK9 that prevents PCSK9‐mediated degradation of hepatic LDL receptors [SG1] [6]. By upregulating LDL‐R recycling, evolocumab dramatically increases LDL clearance [21, 22]. Rosenson et al. note that PCSK9 antibodies in high‐risk groups typically achieve 55%–72% LDL‐C reductions [22]. In our analysis the LDL‐C lowering was comparable, supporting the association between intensive LDL‐C reduction and lower rates of ischemic cardiovascular events, including myocardial infarction and coronary revascularisation.

Our results are consistent with broader PCSK9 inhibitor experience. A recent comprehensive meta‐analysis of all monoclonal PCSK9 inhibitors likewise reported an average LDL‐C decrease of 48 mg/dL (1.24 mmol/L) and significant improvements in total cholesterol, triglycerides, and Lp(a) [23]. In that pooled analysis, PCSK9 inhibitors increased HDL‐C modestly and significantly lowered nonfatal MI, stroke, and revascularisation risk (RR ≈0.87). Our evolocumab‐specific results echo these findings: the reductions in LDL‐C and other lipids were on par with the class effect, and the direction of risk reduction for MI and revascularisation events was similar. In fact, a recent meta‐analysis found that evolocumab alone produced significant reductions in the incidence of MACE, myocardial infarction, stroke, and coronary revascularisation [24]. We likewise observed significant MI and revascularisation risk reductions, while the stroke reduction was directionally favourable but less robust, a pattern seen in earlier trials as well. Notably, our pooled risk ratio for composite MACE favoured evolocumab (RR 0.53; 95% CI 0.40–0.71; *I* ^2^ = 95%), although the magnitude differed from the treatment effect reported in FOURIER (HR approximately 0.85) [5], and the ODYSSEY Outcomes trial of alirocumab (HR ≈0.85) [24, 25]. This difference likely reflects methodological variation, including differences in effect measures, endpoint definitions, follow‐up duration, and study populations.

While our pooled estimate of MACE reduction (RR 0.53) is directionally consistent with prior evidence, its magnitude appears more pronounced than in large event‐driven trials such as FOURIER (HR 0.85) [5], and EVOPACS (RR ~0.84 for 30‐day events) [8]. It is important to note that this pooled estimate is based on risk ratios derived from aggregated event data, whereas key landmark trials such as FOURIER report hazard ratios derived from time‐to‐event survival analyses; therefore, direct numerical comparison between these effect measures should be interpreted with caution. Furthermore, the inclusion of smaller studies, including mechanistic and imaging‐focused trials reporting clinical event outcomes, may contribute to wider uncertainty and potentially amplify relative effect estimates because of smaller event numbers and greater variability. Differences in MACE composition across trials may also influence pooled estimates, as some studies incorporated broader cardiovascular composite definitions including revascularisation or unstable angina, whereas others used more restricted endpoint definitions. Like the inclusion of smaller studies such as Ishihara et al. [15], Kannenkeril et al. [16], and Okada et al. [19], which had fewer absolute cardiovascular events but reported larger relative treatment effects, often over shorter follow‐up durations. These studies, while valid in design, can amplify effect size estimates due to their limited statistical power and higher variability [15, 16, 19]. Additionally, definitions of MACE varied across trials; some included urgent revascularisation or unstable angina [5, 7], while others did not [3, 15, 16, 17, 18, 19, 20], introducing outcome heterogeneity. Therefore, while the pooled result favours Evolocumab for event reduction, we interpret the absolute magnitude of benefit with appropriate caution, noting that the direction of benefit remained generally consistent across studies despite substantial between‐study heterogeneity.

Beyond the established hepatic mechanism of action, additional tissue‐level pathways may contribute to the biological effects observed with PCSK9 inhibition, although these mechanisms remain incompletely understood. The primary mechanism underlying evolocumab‐mediated cardiovascular benefit is increased hepatic LDL receptor availability, resulting in enhanced clearance of circulating LDL particles. However, PCSK9 is also expressed in extrahepatic tissues, including adipose tissue and skeletal muscle, raising the possibility of additional metabolic effects. In adipose tissue, PCSK9 has been implicated in lipid handling, intracellular cholesterol homeostasis, and inflammatory signalling pathways, although the clinical relevance of these effects during pharmacologic PCSK9 inhibition remains uncertain. Similarly, skeletal muscle may be indirectly affected through alterations in cholesterol metabolism; however, unlike statins, PCSK9 inhibitors do not inhibit intracellular cholesterol synthesis, which may contribute to their favourable muscle safety profile observed in randomised trials. Therefore, while hepatic LDL receptor upregulation remains the dominant mechanism explaining cardiovascular benefit, potential extrahepatic effects represent areas for future investigation. The absence of statistically significant reductions in cardiovascular mortality, stroke, and unstable angina in our analysis should be interpreted in the context of endpoint‐specific limitations. Individual cardiovascular outcomes generally occur less frequently than composite MACE and may require longer follow‐up or larger sample sizes to demonstrate statistically robust differences. Furthermore, cardiovascular mortality represents a downstream outcome influenced by multiple competing mechanisms beyond LDL‐C exposure alone, whereas stroke risk may involve additional pathophysiological pathways including atrial fibrillation, hypertension, and cerebrovascular disease burden. Similarly, unstable angina definitions and diagnostic thresholds vary across trials and may be influenced by contemporary revascularisation strategies and clinical practice patterns. Therefore, the absence of statistically significant reductions in these individual endpoints does not necessarily indicate lack of biological benefit, particularly given the consistent reduction observed in myocardial infarction and coronary revascularisation.

Biologically, these cardiovascular benefits are entirely consistent with the ‘lower‐is‐better’ paradigm for LDL‐cholesterol [SG3] [26]. In FOURIER, achieving a median LDL of ~30 mg/dL on evolocumab was associated with steadily fewer events, suggesting that very low LDL targets yield continued benefit [5, 22]. Rosenson et al. even noted that LDL‐C lowering to < 25 mg/dL produced very low event rates, implying no clear safety or efficacy floor [22]. Our meta‐analysis supports this concept: patients on evolocumab achieved exceptionally low LDL‐C levels (often < 30 mg/dL) and accrued proportionately fewer ischemic events. Moreover, evolocumab caused a substantial (~20%–30%) reduction in Lp(a) [10, 27]. The relative contribution of other lipid parameters, including HDL‐C and triglycerides, to clinical outcomes remains less certain. Although these parameters demonstrated statistically significant changes in our pooled analysis, contemporary evidence does not support a causal interpretation of modest HDL‐C elevation or moderate triglyceride reduction as primary mechanisms underlying cardiovascular event reduction with PCSK9 inhibition. Therefore, the observed clinical benefit is most plausibly attributed predominantly to intensive LDL‐C lowering, with potential additional contributions from effects such as Lp(a) reduction. Lp(a) is an independent atherothrombotic risk factor, and its reduction may contribute extra clinical benefit beyond LDL lowering. Mechanistically, the Lp(a) drop can be explained by increased hepatic LDL‐R activity: Lp(a) particles share the LDL receptor pathway, so higher LDL‐R density (from PCSK9 inhibition) augments Lp(a) clearance [10]. A recent study by Rivera FB et al. confirmed that PCSK9 inhibitors reduce Lp(a) by ~27% on average, with evolocumab achieving about a 29% decrease [27]. Our finding of significant Lp(a) lowering therefore aligns with these data, and may have particular importance for patients with elevated baseline Lp(a).

Importantly, evolocumab's efficacy came with an excellent safety and tolerability profile. In pooled analysis there was no excess of serious adverse events compared to control. Injection‐site reactions were slightly more common (as in FOURIER: 2.1% vs. 1.6%) [5], but these were almost all mild. Otherwise, overall adverse event rates (including serious events) were similar to placebo in our meta. Crucially, there was no significant increase in neurocognitive complaints or new‐onset diabetes which mirrors individual trial data and larger PCSK9 meta‐analyses: for example, FOURIER and ODYSSEY reported no differences in rates of cognitive events or incident diabetes [5, 25], and a broad PCSK9 inhibitor review found no uptick in neurological or metabolic toxicity [28]. In our data, neurological and muscle‐related adverse events were not higher with evolocumab, consistent with FOURIER‐OLE, which found no rise in neurocognitive, muscle, hemorrhagic stroke or new‐diabetes events even after 8 years of follow‐up [7]. Drug discontinuations due to side effects were rare. In short, the safety profile of evolocumab was very favourable, effectively indistinguishable from placebo except for the expected minor injection‐site reactions [5, 21].

Substantial heterogeneity was observed across several lipid and clinical outcomes, reflecting differences in study populations, treatment contexts, and trial methodologies rather than inconsistency in the direction of treatment effect. However, statistical significance alone should not be interpreted as evidence of a highly precise treatment estimate when substantial heterogeneity exists. For outcomes with high *I* ^2^ values, including MACE, LDL‐C, triglycerides, HDL‐C, total cholesterol, and Lp(a), the pooled estimates represent average effects across heterogeneous study settings, and the true magnitude of benefit may differ according to patient characteristics and clinical context. For MACE specifically, the high heterogeneity (*I* ^2^ = 95%) indicates that the magnitude of the pooled estimate varied substantially across studies, despite a generally consistent direction of benefit. Therefore, the pooled RR should be interpreted primarily as evidence supporting a favourable direction of effect rather than as a precise estimate of the absolute magnitude of cardiovascular risk reduction achievable with evolocumab. Variability in baseline LDL‐C concentrations may have influenced the magnitude of absolute lipid reductions, as patients with higher pretreatment LDL‐C levels may experience greater absolute reductions following PCSK9 inhibition. Additionally, differences in background lipid‐lowering therapy, including intensity of statin therapy and concomitant use of other lipid‐modifying agents, may have contributed to variability in observed treatment effects across studies. The clinical characteristics of enrolled populations may also explain heterogeneity, as trials including patients with acute coronary syndrome represent a biologically distinct population compared with stable ASCVD cohorts, with differences in inflammatory status, plaque instability, baseline cardiovascular risk, and treatment timing. Furthermore, variability in trial objectives and designs likely contributed to between‐study differences. Event‐driven cardiovascular outcome trials were designed to evaluate long‐term clinical endpoints, whereas lipid‐focused, mechanistic, and imaging studies primarily assessed surrogate markers over shorter or intermediate follow‐up periods. Differences in endpoint definitions, follow‐up duration, baseline risk profiles, and study‐specific treatment protocols may therefore influence pooled estimates. The persistence of residual heterogeneity despite sensitivity and subgroup analyses suggests that additional unmeasured factors may influence treatment response. These may include baseline cardiovascular risk, intensity of background statin therapy, timing of evolocumab initiation relative to cardiovascular events, baseline LDL‐C and Lp(a) concentrations, and differences in endpoint adjudication. Therefore, while the overall direction of benefit was consistent, the precise magnitude of treatment effect should be interpreted within the context of individual patient risk profiles and clinical settings.

The certainty of evidence assessment provides additional context for interpreting these findings. Although the overall direction of benefit with evolocumab was consistent across outcomes, confidence in individual estimates varied according to the certainty of available evidence. High‐certainty evidence supported reductions in myocardial infarction and coronary revascularisation, as well as the absence of excess serious adverse events, strengthening confidence in these clinically important outcomes. In contrast, several outcomes, including LDL‐C reduction, major adverse cardiovascular events, cardiovascular mortality, and lipid parameters, were supported by moderate‐certainty evidence, primarily because of between‐study heterogeneity and differences in trial populations, designs, and follow‐up durations. Therefore, while these findings support the cardiovascular efficacy of evolocumab, the exact magnitude of benefit, particularly for composite outcomes such as MACE, should be interpreted with appropriate caution. The certainty of evidence for lipoprotein(a) reduction was rated as low due to substantial heterogeneity and imprecision, indicating that although evolocumab lowers lipoprotein(a), the clinical implications of this effect remain uncertain. Incorporating certainty‐of‐evidence assessments alongside effect estimates allows a more balanced interpretation of the available evidence and supports individualised clinical decision‐making based on both treatment efficacy and confidence in the estimated benefit.

### 4.1 Strength and Limitations

Several strengths of this meta‐analysis merit emphasis. First, the pooled sample is very large (nearly 68,000 patients), providing high statistical power to detect even moderate differences in events and safety outcomes. This far exceeds individual trials and thus allows precise estimates of rare harms (e.g., neurocognitive events) that smaller RCTs cannot discern. Second, we focused exclusively on evolocumab rather than lumping all PCSK9 inhibitors together. This isolates drug‐specific effects and avoids confounding by alirocumab differences. Third, all included studies were randomised controlled trials with adjudicated outcomes, which minimises bias. Finally, the consistency of the direction of treatment effects across diverse trials (Phase 2–3 studies, short‐term and long‐term follow‐up) lends confidence that the results are robust.

Nevertheless, this analysis has limitations. There was heterogeneity among trials in design and duration. Follow‐up ranged from a few months in short‐term lipid studies to ~3 years in outcome trials, which may attenuate observed event reductions in shorter studies. Some smaller trials were lipid‐only (no clinical endpoints), which dilutes the overall MACE effect; we included them to comprehensively assess lipid and safety changes [19]. Variability in patient populations (acute coronary syndrome vs. stable CAD, differing statin regimens) also introduces heterogeneity—for example, EVOPACS enrolled post‐ACS patients, whereas FOURIER studied stable CVD [5, 8]. Furthermore, because this analysis was based on aggregate study‐level data rather than individual participant‐level data, we were unable to evaluate whether specific patient characteristics modified the magnitude of benefit associated with evolocumab. Important clinical factors, including age, sex, diabetes status, renal function, inflammatory status, baseline LDL‐C concentration, adherence to background statin therapy, and concomitant lipid‐lowering medications, could not be consistently adjusted for across studies because of incomplete or heterogeneous reporting. Consequently, the pooled estimates should be interpreted as average treatment effects across trial populations rather than patient‐specific estimates, and future individual patient‐level meta‐analyses are needed to better define populations most likely to derive the greatest benefit. Furthermore, differences in baseline LDL‐C concentrations, intensity of background statin therapy, and concomitant lipid‐lowering medications may have influenced the magnitude of treatment effects; however, these individual‐level factors were inconsistently reported across trials and could not be fully evaluated. We attempted subgroup analyses where possible, but the aggregate data nature precludes patient‐level adjustment. Outcome definitions of MACE were not identical across trials (e.g., some included unstable angina or urgent revascularisation differently), which could introduce measurement bias. Another limitation is follow‐up duration: most trials were relatively short (2–3 years); we thus rely on extension data for very long‐term inference [7]. Additionally, differences in effect measures (RR in this meta‐analysis versus HR in major individual trials) may partially contribute to apparent differences in effect magnitude and heterogeneity for MACE. Although subgroup analyses were conducted to explore sources of heterogeneity, these analyses were exploratory in nature and limited by incomplete and inconsistent reporting of key clinical and methodological variables across included trials. Importantly, the presence of statistical significance in random‐effects models does not eliminate uncertainty arising from between‐study variability. Therefore, our findings should be interpreted as demonstrating evidence of benefit with evolocumab while acknowledging that the magnitude of benefit may vary across different patient populations and healthcare settings. Finally, all included studies were industry‐funded, raising the possibility of publication bias or selective reporting. We did not formally assess publication bias for rare safety endpoints.

### 4.2 Clinical Implications

Clinically, these findings reinforce current guideline recommendations to use PCSK9 inhibitors in very‐high‐risk patients who have not reached LDL goals on statins and ezetimibe [2, 22]. Specifically, the 2018 ACC/AHA consensus and 2019 ESC/EAS guidelines both set an LDL‐C threshold (~70 mg/dL) above which PCSK9 blockade is advised in ASCVD patients [2, 22]. Our meta‐analysis shows that adding evolocumab in such patients not only achieves guideline LDL targets (often pushing levels far below 70 mg/dL) but also is associated with additional reductions in major adverse cardiovascular events (MACE) and specific ischemic cardiovascular endpoints, particularly myocardial infarction and coronary revascularisation. Although substantial heterogeneity was observed across several efficacy outcomes, the overall direction of benefit was consistent with current evidence from major randomised trials. The observed benefits support consideration of evolocumab for eligible patients. However, practical implementation will hinge on cost and access. Importantly, the magnitude of clinical benefit with evolocumab is expected to vary according to baseline cardiovascular risk rather than being uniform across all patients with ASCVD. While relative risk reductions provide an estimate of treatment efficacy, absolute risk reduction and the number needed to treat are strongly influenced by baseline event rates. Therefore, patients with the highest residual cardiovascular risk, including those with recent acute coronary syndrome, multiple prior cardiovascular events, polyvascular disease, diabetes, chronic kidney disease, persistently elevated LDL‐C despite maximally tolerated lipid‐lowering therapy, or markedly elevated lipoprotein(a) concentrations, are more likely to derive greater absolute benefit from additional PCSK9 inhibition. This principle is consistent with contemporary approaches to personalised cardiovascular prevention, where treatment intensity is tailored according to both residual risk and expected absolute risk reduction rather than relative treatment effects alone. Current drug prices remain high, and earlier cost‐effectiveness analyses have been unfavourable. Recent systematic reviews suggest that evolocumab can be cost‐effective primarily in the highest‐risk subgroups (e.g., multiple prior events) [29]. In practice, insurers often require demonstration of very high LDL or multiple cardiovascular events before approval, reflecting the need to maximise absolute benefit [30, 31].

Our findings support such targeted use: the more patients fit the very‐high‐risk profile (e.g., recent ACS, polyvascular disease, markedly elevated LDL‐C or Lp(a)), the greater the potential absolute risk reduction with evolocumab. Beyond residual LDL‐C elevation, contemporary ASCVD management increasingly recognises persistent inflammatory and lipoprotein(a)‐mediated risk as contributors to recurrent cardiovascular events despite optimised lipid therapy. Although evolocumab does not directly target inflammatory pathways, its modest reduction in Lp(a), together with intensive LDL‐C lowering, may provide additional benefit in selected patients with elevated residual atherothrombotic risk. However, the extent to which Lp(a) lowering independently contributes to cardiovascular event reduction remains uncertain and requires further dedicated investigation. In the future, price reductions and new value‐based pricing may expand access. Health systems should weigh the substantial clinical gains (prevented MIs and strokes) against cost; our results provide quantitative estimates to inform such decisions.

### 4.3 Future Research

Looking ahead, several areas warrant further study. Longer‐term outcomes beyond 3–5 years remain of interest. The FOURIER‐OLE data up to 8 years show durable benefit and safety [7], but real‐world long‐term registries would help confirm these findings outside trial settings. Subgroup analyses could also be informative: for example, patients with baseline Lp(a) above 50 mg/dL might derive particular benefit from evolocumab (via its Lp(a)‐lowering effect), but dedicated studies or pooled patient‐level analyses are needed. Similarly, whether certain comorbidities (such as chronic kidney disease or frailty) modify risk–benefit is still unclear [28]. Head‐to‐head comparisons between PCSK9 inhibitors (evolocumab vs. alirocumab) might clarify if one agent has superior efficacy or tolerability; network meta‐analyses hint that evolocumab may rank slightly higher for LDL reduction [32]. Cost‐effectiveness must be revisited as drug pricing and healthcare economics evolve [29]. Future studies should prioritise individual participant data meta‐analyses to identify patient characteristics associated with greater clinical benefit from evolocumab, including baseline LDL‐C, lipoprotein(a) levels, diabetes status, and residual inflammatory risk. Comparative effectiveness studies evaluating evolocumab against newer lipid‐lowering strategies, including inclisiran and emerging lipoprotein(a)‐targeted therapies, are also needed. Additionally, investigations into cost‐effectiveness across diverse healthcare systems and biomarkers predictive of response may help refine patient selection and optimise personalised PCSK9 inhibitor therapy. As novel therapies such as PCSK9‐silencing approaches and Lp(a)‐targeted agents continue to develop, future comparative studies will be needed to determine their optimal integration with established PCSK9 monoclonal antibody therapy [32].

## 5 Conclusion

Our meta‐analysis provides evidence, with varying certainty across outcomes, that evolocumab, added to standard lipid therapy in patients with established CVD, achieves very high‐intensity LDL lowering and reduces ischemic cardiovascular events without compromising safety. Although the pooled MACE estimate was stronger than previously reported in large trials, this likely reflects the contribution of smaller studies with shorter follow‐up and fewer absolute events. Accordingly, the pooled estimate should not be interpreted as a direct measure of a 50% reduction in cardiovascular events, but rather as supportive evidence of cardiovascular benefit consistent with the broader randomised trial literature. The cardiovascular benefit of evolocumab remains biologically and clinically plausible, and directionally consistent with guideline‐based expectations. These results corroborate the current understanding of PCSK9 biology and reinforce the principle that intensive LDL‐C lowering improves outcomes. The large pooled sample and focus on evolocumab strengthen the precision and relevance of our findings. Although substantial heterogeneity was observed across several efficacy outcomes, the overall direction of benefit remained consistent across studies. Nevertheless, these findings should not be interpreted as indicating an identical magnitude of treatment effect across all populations, as residual heterogeneity suggests that the clinical impact of evolocumab may depend on patient characteristics, baseline risk, and study‐specific factors. Clinicians may consider the use of evolocumab in appropriate high‐risk patients, given its association with meaningful improvements in lipid profiles and favourable cardiovascular outcomes, while maintaining vigilance for known mild side effects such as injection‐site reactions. Future work should aim to extend follow‐up, refine patient selection, and evaluate real‐world cost‐effectiveness, but the existing evidence supports evolocumab as an efficacious and generally safe therapy for secondary prevention in CVD.

## Author Contributions

**Anika Goel:** conceptualization, methodology, formal analysis. **Muhammad Abdullah Naveed:** methodology, formal analysis, writing – original draft. **Hemeesh Tandel:** methodology, formal analysis, writing – original draft. **Affaf Mahmood:** writing – review and editing, validation, data curation, investigation. **Muhammad Mukhlis:** visualization, writing – original draft, writing – review and editing. **K. Sai Varun Reddy:** investigation, validation, data curation. **Arun Kumar Maloth:** data curation, validation, writing – review and editing. **Anchit Chauhan:** data curation, writing – review and editing, resources. **Ameer Ashwara:** data curation, writing – review and editing, validation. **Amogh Verma:** investigation, writing – review and editing, supervision. **Haris Bin Tahir:** supervision, project administration, visualization, writing – review and editing.

## Funding

The authors have nothing to report.

## Ethics Statement

The authors have nothing to report.

## Consent

The authors have nothing to report.

## Conflicts of Interest

The authors declare no conflicts of interest.

## Supporting information


**Table S1:** Search strategy.
**Table S2:** Certainty of evidence for outcomes assessed using the GRADE approach.
**Table S3:** Leave‐one‐out sensitivity analysis evaluating the contribution of individual studies to between‐study heterogeneity for efficacy and safety outcomes.
**Figure S1:** Forest plot showing changes in Lipoprotein (a) Levels.
**Figure S2:** Forest plot showing Major Adverse Cardiovascular Events (MACE).
**Figure S3:** Forest plot showing Cardio‐vascular deaths.
**Figure S4:** Forest plot showing hospitalisations for unstable angina.
**Figure S5:** Forest plot showing the outcome of stroke.
**Figure S6:** Forest plot showing Myocardial Infarction (MI).
**Figure S7:** Forest plot showing coronary revascularisation.
**Figure S8:** Forest plot showing Serious Adverse events.
**Figure S9:** Forest plot showing drug discontinuation.
**Figure S10:** Forest plot showing neurocognitive events.
**Figure S11:** Forest plot showing injection site reactions.
**Figure S12:** Forest plot showing muscle related events.
**Figure S13:** Forest plot of Sensitivity Analysis of Total Cholesterol.
**Figure S14:** Forest plot of Sensitivity Analysis of Triglycerides.
**Figure S15:** Forest plot of Sensitivity Analysis of HDL.
**Figure S16:** Forest plot of Sensitivity Analysis of LDL.
**Figure S17:** Forest plot of Sensitivity Analysis of LP (a).
**Figure S18:** Forest plot of Sensitivity Analysis of Drug Discontinuation.
**Figure S19:** Forest plot of Sensitivity Analysis of Injection Site Reaction.
**Figure S20:** Forest plot of Sensitivity Analysis of MACE after excluding Sabatline 2015.
**Figure S21:** Forest plot of Sensitivity Analysis of MACE after removing small studies Nicholis 2016 and 2022.
**Figure S22:** Forest plot of Sensitivity Analysis of MACE after removing Nicholis 2016, 2022 and Sabatline 2015.
**Figure S23:** Forest plot of subgroup analysis of total cholesterol in ACS versus stable CAD populations.
**Figure S24:** Forest plot of subgroup analysis of total cholesterol according to outcome‐based trial design.
**Figure S25:** Forest plot of subgroup analysis of total cholesterol according to follow‐up duration.
**Figure S26:** Forest plot of subgroup analysis of LDL‐C in ACS versus stable CAD populations.
**Figure S27:** Forest plot of subgroup analysis of LDL‐C according to outcome‐based trial design.
**Figure S28:** Forest plot of subgroup analysis of LDL‐C according to follow‐up duration.
**Figure S29:** Forest plot of subgroup analysis of triglycerides in ACS versus stable CAD populations.
**Figure S30:** Forest plot of subgroup analysis of triglycerides according to outcome‐based trial design.
**Figure S31:** Forest plot of subgroup analysis of triglycerides according to follow‐up duration.
**Figure S32:** Forest plot of subgroup analysis of HDL in ACS versus stable CAD populations.
**Figure S33:** Forest plot of subgroup analysis of HDL according to outcome‐based trial design.
**Figure S34:** Forest plot of subgroup analysis of HDL according to follow‐up duration.
**Figure S35:** Forest plot of subgroup analysis of lipoprotein (a) in ACS versus stable CAD populations.
**Figure S36:** Forest plot of subgroup analysis of lipoprotein (a) according to outcome‐based trial design.
**Figure S37:** Forest plot of subgroup analysis of lipoprotein (a) according to follow‐up duration.
**Figure S38:** Forest plot of subgroup analysis of major adverse cardiovascular events (MACE) in ACS versus stable CAD populations.
**Figure S39:** Forest plot of subgroup analysis of MACE according to outcome‐based trial design.
**Figure S40:** Forest plot of subgroup analysis of MACE according to follow‐up duration.
**Figure S41:** Forest plot of subgroup analysis of treatment discontinuation in ACS versus stable CAD populations.
**Figure S42:** Forest plot of subgroup analysis of treatment discontinuation according to outcome‐based trial design.
**Figure S43:** Forest plot of subgroup analysis of injection‐site reactions in ACS versus stable CAD populations.
**Figure S44:** Forest plot of subgroup analysis of injection‐site reactions according to outcome‐based trial design.
**Figure S45:** Forest plot of subgroup analysis of injection‐site reactions according to follow‐up duration.

## Data Availability

All data generated or analysed during this study are included in the manuscript and its Supporting Information. Additional details are available from the corresponding author on reasonable request.
