# Supplementary material for: Impact of Evolocumab on LDL‐Cholesterol and Cardiovascular Outcomes in Patients With Established CVD: Systematic Review and Meta‐Analysis
Source: Endocrinol Diabetes Metab. 2026 Oct 8;9(6):e70355. doi: 10.1002/edm2.70355 (PMC13650179; doi:10.1002/edm2.70355)
Supplement: Supplementary file 1 [file EDM2-9-e70355-s001.docx]

**Table S1.** Search strategy.

| **Database** | **Search Terms** | **Search Results** |
| --- | --- | --- |
| Pubmed | ("evolocumab"[MeSH] OR "evolocumab") AND  ("cardiovascular diseases"[MeSH] OR "myocardial infarction"[MeSH] OR  "stroke"[MeSH] OR "peripheral arterial disease"[MeSH] OR  "coronary artery disease"[MeSH] OR "coronary revascularization" OR  "atherosclerosis"[MeSH]) AND  ("randomized controlled trial"[Publication Type] OR "randomized"[Title/Abstract] OR  "clinical trial"[Publication Type] OR "post hoc"[Title/Abstract] OR "secondary analysis"[Title/Abstract] OR "subgroup analysis"[Title/Abstract]) AND  "humans"[MeSH] | 469 |
| Cochrane | (evolocumab):ti,ab,kw AND  (cardiovascular OR myocardial infarction OR stroke OR coronary revascularization OR peripheral arterial disease):ti,ab,kw AND  (random* OR placebo* OR "post hoc" OR "secondary analysis" OR "subgroup analysis"):ti,ab,kw IN Trials | 353 |
| Clinical Trials | Evolocumab AND (cardiovascular OR "myocardial infarction" OR stroke OR "peripheral arterial disease" OR "coronary revascularization") AND  ("post hoc" OR "secondary analysis" OR "extension study" OR "subgroup analysis") AND  (interventional) AND (Phase 2 OR Phase 3) | 182 |
| SCOPUS | (TITLE-ABS-KEY(evolocumab)) AND  (TITLE-ABS-KEY("cardiovascular disease" OR "myocardial infarction" OR "stroke" OR  "peripheral arterial disease" OR "coronary artery disease" OR "atherosclerosis" OR  "coronary revascularization")) AND  (TITLE-ABS-KEY("randomized controlled trial" OR "clinical trial" OR randomized OR placebo  OR "post hoc analysis" OR "secondary analysis" OR "subgroup analysis")) | 234 |
| Embase | ('evolocumab'/exp OR 'evolocumab') AND  ('cardiovascular disease'/exp OR 'myocardial infarction'/exp OR  'stroke'/exp OR 'peripheral arterial disease'/exp OR 'coronary revascularization'/exp) AND  ('randomized controlled trial'/exp OR 'randomized controlled trial' OR 'randomization' OR  'post hoc analysis' OR 'secondary analysis' OR 'subgroup analysis') AND  [humans]/lim AND [english]/lim | 173 |

**Table S2.** Certainty of Evidence for Outcomes Assessed Using the GRADE Approach

| **Outcome** | **No. of Included Studies** | **Relative Effect (RR/MD, 95% CI)** | **Certainty of the Evidence (GRADE)** |
| --- | --- | --- | --- |
| **Efficacy Outcomes** |  |  |  |
| Total Cholesterol | 5 studies | MD −52.27 mg/dL (−66.66 to −37.89) | ⊕⊕⊕◯ Moderateᵃ |
| Triglycerides | 6 studies | MD −21.21 mg/dL (−26.03 to −16.38) | ⊕⊕⊕◯ Moderateᵇ |
| HDL-C | 6 studies | MD 3.69 mg/dL (1.70 to 5.67) | ⊕⊕⊕◯ Moderateᶜ |
| LDL-C | 5 studies | MD −57.73 mg/dL (−60.67 to −54.78) | ⊕⊕⊕◯ Moderateᵈ |
| Lipoprotein(a) [Lp(a)] | 5 studies | MD −10.99 mg/dL (−22.89 to 0.90) | ⊕⊕◯◯ Lowᵉ |
| Major Adverse Cardiovascular Events (MACE) | 5 studies | RR 0.53 (0.40 to 0.71) | ⊕⊕⊕◯ Moderateᶠ |
| Cardiovascular Mortality | 5 studies | RR 0.92 (0.79 to 1.07) | ⊕⊕⊕◯ Moderateᵍ |
| Stroke | 5 studies | RR 0.86 (0.74 to 1.00) | ⊕⊕⊕⊕ Highʰ |
| Myocardial Infarction | 5 studies | RR 0.74 (0.67 to 0.82) | ⊕⊕⊕⊕ Highⁱ |
| Coronary Revascularization | 5 studies | RR 0.80 (0.74 to 0.86) | ⊕⊕⊕⊕ Highʲ |
| **Safety Outcomes** |  |  |  |
| Serious Adverse Events | 6 studies | RR 1.00 (0.97 to 1.03) | ⊕⊕⊕⊕ Highᵏ |
| Drug Discontinuation Due to Adverse Events | 3 studies | RR 0.95 (0.74 to 1.22) | ⊕⊕⊕◯ Moderateˡ |
| Neurocognitive Adverse Events | 4 studies | RR 1.12 (0.94 to 1.32) | ⊕⊕⊕◯ Moderateᵐ |
| Injection-Site Reactions | 5 studies | RR 1.56 (0.88 to 2.75) | ⊕⊕⊕◯ Moderateⁿ |
| Muscle-Related Adverse Events | 4 studies | RR 1.05 (0.97 to 1.15) | ⊕⊕⊕◯ Moderateᵒ |

**Note:** The GRADE Working Group defines certainty of evidence as follows:

**High certainty:** We are highly confident that the actual effect is very close to the estimated effect.

**Moderate certainty:** We are fairly confident in the estimate; the true effect is likely similar to the estimate, but there remains a possibility that it is substantially different.

**Low certainty:** Our confidence in the estimate is limited, and the actual effect may be substantially different from the estimated effect.

**Very low certainty:** We have very little confidence in the estimate, and the true effect is likely substantially different from the estimated effect.

ᵃ The certainty of evidence for total cholesterol was downgraded by one level for inconsistency due to considerable heterogeneity across studies (I² = 100%).

ᵇ The certainty of evidence for triglycerides was downgraded by one level for inconsistency due to substantial heterogeneity across studies (I² = 92%).

ᶜ The certainty of evidence for HDL-C was downgraded by one level for inconsistency due to considerable heterogeneity across studies (I² = 98%).

ᵈ The certainty of evidence for LDL-C was downgraded by one level for inconsistency due to substantial heterogeneity across studies (I² = 91%).

ᵉ The certainty of evidence for Lp(a) was downgraded by one level for inconsistency due to considerable heterogeneity (I² = 99%) and by one level for imprecision because the confidence interval crossed the line of no effect.

ᶠ The certainty of evidence for MACE was downgraded by one level for inconsistency due to considerable heterogeneity across studies (I² = 95%), although the direction of effect remained consistent.

ᵍ The certainty of evidence for cardiovascular mortality was downgraded by one level for imprecision because the confidence interval crossed the line of no effect.

ʰ The certainty of evidence for stroke was rated as high because evidence was derived from randomized controlled trials with low risk of bias, minimal heterogeneity (I² = 5%), and a consistent direction of effect.

ⁱ The certainty of evidence for myocardial infarction was rated as high because evidence was derived from randomized controlled trials with low risk of bias, no observed heterogeneity (I² = 0%), and precise effect estimates.

ʲ The certainty of evidence for coronary revascularization was rated as high because evidence was derived from randomized controlled trials with low risk of bias, low heterogeneity (I² = 31%), and precise effect estimates.

ᵏ The certainty of evidence for serious adverse events was rated as high because evidence was derived from randomized controlled trials with low risk of bias, no observed heterogeneity (I² = 0%), and narrow confidence intervals.

ˡ The certainty of evidence for drug discontinuation due to adverse events was downgraded by one level for inconsistency and imprecision due to substantial heterogeneity (I² = 70%) and confidence intervals crossing the line of no effect.

ᵐ The certainty of evidence for neurocognitive adverse events was downgraded by one level for imprecision because the confidence interval crossed the line of no effect.

ⁿ The certainty of evidence for injection-site reactions was downgraded by one level for inconsistency and imprecision due to substantial heterogeneity (I² = 67%) and confidence intervals crossing the line of no effect.

ᵒ The certainty of evidence for muscle-related adverse events was downgraded by one level for imprecision because the confidence interval crossed the line of no effect despite low statistical heterogeneity (I² = 0%).

| ***Outcome*** | ***No of studies*** | ***Initial I²***  ***(%)*** | ***Initial p value*** | ***Study removed*** | ***New I² (%)*** | ***New P value*** |
| --- | --- | --- | --- | --- | --- | --- |
| ***Total Cholesterol*** | 5 | 100 | <0.00001 | Deedwania 2020 | 0 | <0.00001 |
| ***Triglycerides*** | 6 | 92 | <0.00001 | Ishihara 2022 | 63 | <0.00001 |
| ***HDL*** | 6 | 98 | 0.0003 | Nicholis 2016 | 89 | <0.00001 |
| ***LDL*** | 5 | 91 | <0.000001 | O'Donoghue 2022 | 0 | <0.00001 |
| ***LP(a)*** | 5 | 99 | 0.07 | Nicholis 2016 | 98 | 0.01 |
|  |  |  |  |  |  |  |
| ***Drug Discontinuation*** | 3 | 70 | 0.67 | Sabatline 2017 | 46 | 0.35 |
| ***Injection Site Reaction*** | 5 | 67 | 0.13 | Sabatline 2015 | 0 | 0.0004 |
| ***MACE*** | | | | | | |
| ***After Excluding only One Study*** | 5 | 95 | 0.0001 | Sabatline 2015 | 88 | 0.0006 |
| ***After Excluding only Two Study*** | 5 | 95 | 0.0001 | Nicholis 2016 and 2022 | 97 | 0.0004 |
| ***After Excluding only Thre Study*** | 5 | 95 | 0.0001 | Nicholis 2016, 2022 and Sabatline 2015. | 0 | 0.00001 |

**Table S3.**  Leave-one-out sensitivity analysis evaluating the contribution of individual studies to between-study heterogeneity for efficacy and safety outcomes.

**Figure S1.** Forest plot showing changes in Lipoprotein (a) Levels.

**Figure S2.** Forest plot showing Major Adverse Cardiovascular Events (MACE).

**Figure S3.** Forest plot showing Cardio-vascular deaths.

**Figure S4.** Forest plot showing hospitalisations for unstable angina.

**Figure S5.** Forest plot showing the outcome of stroke.

**Figure S6.** Forest plot showing Myocardial Infarction (MI).

**Figure S7.** Forest plot showing coronary revascularisation.

**Figure S8.** Forest plot showing Serious Adverse events.

**Figure S9.** Forest plot showing drug discontinuation.

**Figure S10.** Forest plot showing neurocognitive events.

**Figure S11.** Forest plot showing injection site reactions.

**Figure S12.** Forest plot showing muscle related events.

**Figure S13.** Forest plot of Sensitivity Analysis of Total Cholesterol.

**Figure S14.** Forest plot of Sensitivity Analysis of Triglycerides.

**Figure S15.** Forest plot of Sensitivity Analysis of HDL.

**Figure S16.** Forest plot of Sensitivity Analysis of LDL.

 **Figure S17.** Forest plot of Sensitivity Analysis of LP (a).

**Figure S18.** Forest plot of Sensitivity Analysis of Drug Discontinuation.

**Figure S19.** Forest plot of Sensitivity Analysis of Injection Site Reaction.

**Figure S20.** Forest plot of Sensitivity Analysis of MACE after excluding Sabatline 2015.

**Figure S21.** Forest plot of Sensitivity Analysis of MACE after removing small studies Nicholis 2016 and 2022.

**Figure S22.** Forest plot of Sensitivity Analysis of MACE after removing Nicholis 2016, 2022 and Sabatline 2015.

**Subgroup Analysis:**

To explore potential sources of heterogeneity and address concerns regarding the robustness of pooled estimates, prespecified subgroup analyses were performed for outcomes demonstrating substantial heterogeneity (I² > 50%). Prespecified subgroup analyses were not included in the original study registration but were conceptually planned during protocol development based on the anticipated clinical heterogeneity of the included populations. During the analysis phase, limitations in available study-level data and inconsistent reporting across trials restricted the feasibility of conducting all planned subgroup comparisons. Consequently, subgroup analyses were performed in an exploratory, hypothesis-generating manner and should be interpreted with caution. Subgroups were stratified according to clinical presentation (acute coronary syndrome [ACS] versus stable coronary artery disease/atherosclerotic cardiovascular disease [ASCVD]), trial design (event-driven trials, lipid-only trials, mechanistic trials, and imaging/atherosclerosis structure trials), and follow-up duration (<1 year versus ≥1 year). Event-driven trials were defined as studies primarily designed to assess clinical cardiovascular outcomes, whereas lipid-only trials focused predominantly on lipid parameter changes. Mechanistic trials investigated physiological or biological effects of Evolocumab, while imaging/atherosclerosis structure trials evaluated changes in plaque burden, atherosclerotic progression, or vascular morphology using imaging modalities. These analyses were conducted to identify potential contributors to between-study heterogeneity and assess the consistency of treatment effects across clinically relevant study characteristics.

**Total Cholesterol:**

For total cholesterol, subgroup analysis based on clinical presentation demonstrated a significant reduction in total cholesterol levels in both ACS and stable CAD/ASCVD populations. In ACS trials (2 studies), Evolocumab significantly reduced total cholesterol levels (MD = −59.31 mg/dL, 95% CI: −69.82 to −48.79; P < 0.00001), with no observed heterogeneity (I² = 0%). Similarly, studies involving patients with stable CAD/ASCVD (3 studies) demonstrated a significant reduction in total cholesterol (MD = −48.48 mg/dL, 95% CI: −66.57 to −30.38; P < 0.00001). However, substantial heterogeneity persisted within the stable CAD/ASCVD subgroup (I² = 100%) (Figure S23).

**Figure S23: Forest plot of subgroup analysis of total cholesterol in ACS versus stable CAD populations.**

Subgroup analysis according to trial design demonstrated consistent reductions in total cholesterol across all study categories. The single event-driven trial reported a significant reduction in total cholesterol (MD = −35.30 mg/dL, 95% CI: −36.08 to −34.52; P < 0.00001). Likewise, the lipid-only trial demonstrated a significant decrease (MD = −53.50 mg/dL, 95% CI: −69.14 to −37.86; P < 0.00001). Imaging/atherosclerosis structure trials (2 studies) also showed a significant reduction in total cholesterol (MD = −57.20 mg/dL, 95% CI: −57.45 to −56.96; P < 0.00001) with no heterogeneity (I² = 0%). The mechanistic trial similarly demonstrated a significant reduction (MD = −54.00 mg/dL, 95% CI: −68.94 to −39.06; P < 0.00001) (Figure S24).

**Figure S24: Forest plot of subgroup analysis of total cholesterol according to outcome-based trial design.**

When stratified by follow-up duration, studies with follow-up periods of less than 1 year (2 studies) demonstrated a significant reduction in total cholesterol (MD = −53.76 mg/dL, 95% CI: −64.57 to −42.96; P < 0.00001), with no heterogeneity (I² = 0%). Studies with follow-up durations greater than 1 year (3 studies) also showed a significant reduction in total cholesterol (MD = −51.44 mg/dL, 95% CI: −69.49 to −33.39; P < 0.00001); however, considerable heterogeneity remained (I² = 100%) (Figure S25). Overall, the magnitude and direction of effect remained consistent across all subgroups, indicating that the cholesterol-lowering effect of Evolocumab was robust irrespective of clinical presentation, trial design, or follow-up duration. Nevertheless, persistent heterogeneity within the stable CAD/ASCVD and longer follow-up subgroups suggests that additional study-level factors may contribute to the observed variability.

**Figure S25: Forest plot of subgroup analysis of total cholesterol according to follow-up duration.**

**LDL:**

For LDL-C, subgroup analysis based on clinical presentation demonstrated a significant reduction in LDL-C levels across both ACS and stable CAD/ASCVD populations. The ACS subgroup, represented by a single study, showed a substantial reduction in LDL-C levels with Evolocumab (MD = −58.90 mg/dL, 95% CI: −72.64 to −45.16; P < 0.00001). Similarly, studies involving patients with stable CAD/ASCVD (4 studies) demonstrated a significant reduction in LDL-C levels (MD = −57.67 mg/dL, 95% CI: −60.71 to −54.63; P < 0.00001). However, considerable heterogeneity remained within the stable CAD/ASCVD subgroup (I² = 93%) (Figure S26).

**Figure S26: Forest plot of subgroup analysis of LDL-C in ACS versus stable CAD populations.**

Subgroup analysis according to trial design demonstrated consistent LDL-C reductions across all study categories. The single event-driven trial reported a significant reduction in LDL-C levels (MD = −60.00 mg/dL, 95% CI: −61.04 to −58.96; P < 0.00001). Mechanistic trials (2 studies) also demonstrated a significant reduction in LDL-C (MD = −55.51 mg/dL, 95% CI: −63.98 to −47.03; P < 0.00001), with moderate heterogeneity (I² = 58%). Likewise, imaging/atherosclerosis structure trials (2 studies) showed a significant reduction in LDL-C levels (MD = −56.34 mg/dL, 95% CI: −56.77 to −55.91; P < 0.00001) with no heterogeneity (I² = 0%) (Figure S27).

**Figure S27: Forest plot of subgroup analysis of LDL-C according to outcome-based trial design.**

When stratified by follow-up duration, studies with follow-up periods of less than 1 year (2 studies) demonstrated a significant reduction in LDL-C levels (MD = −55.51 mg/dL, 95% CI: −63.98 to −47.03; P < 0.00001), with moderate heterogeneity (I² = 58%). Studies with follow-up durations greater than 1 year (3 studies) similarly demonstrated a significant reduction in LDL-C levels (MD = −58.18 mg/dL, 95% CI: −61.62 to −54.74; P < 0.00001); however, substantial heterogeneity persisted (I² = 95%) (Figure 28). Overall, the LDL-C lowering effect of Evolocumab remained highly consistent across all subgroup analyses, with comparable effect sizes irrespective of clinical presentation, study design, or follow-up duration. The reduction of heterogeneity in imaging trials and partial reduction in mechanistic and short-term follow-up studies suggests that differences in study characteristics may explain some, but not all, of the variability observed in the overall analysis.

**Figure S28: Forest plot of subgroup analysis of LDL-C according to follow-up duration.**

**Triglycerides:**

For triglycerides (TAGs), subgroup analysis based on clinical presentation demonstrated significant reductions in TAG levels among both ACS and stable CAD/ASCVD populations. In ACS trials (2 studies), Evolocumab significantly reduced TAG levels (MD = −19.00 mg/dL, 95% CI: −19.54 to −18.45; P < 0.00001), with no observed heterogeneity (I² = 0%). Similarly, studies involving patients with stable CAD/ASCVD (4 studies) demonstrated a significant reduction in TAG levels (MD = −22.71 mg/dL, 95% CI: −34.30 to −11.12; P = 0.0001). However, substantial heterogeneity remained within the stable CAD/ASCVD subgroup (I² = 95%) (Figure S29).

**Figure S29: Forest plot of subgroup analysis of triglycerides in ACS versus stable CAD populations.**

Subgroup analysis according to trial design showed largely consistent reductions in TAG levels across study categories. The single event-driven trial demonstrated a significant reduction in TAG levels (MD = −16.10 mg/dL, 95% CI: −17.84 to −14.36; P < 0.00001). In contrast, the lipid-only trial did not demonstrate a statistically significant effect (MD = −9.30 mg/dL, 95% CI: −46.37 to 27.77; P = 0.62). Mechanistic trials (2 studies) showed a significant reduction in TAG levels (MD = −29.83 mg/dL, 95% CI: −32.78 to −26.88; P < 0.00001) with no heterogeneity (I² = 0%). Similarly, imaging/atherosclerosis structure trials demonstrated a significant reduction in TAG levels (MD = −19.00 mg/dL, 95% CI: −19.54 to −18.45; P < 0.00001), also with no heterogeneity (I² = 0%) (Figure S30).

**Figure S30: Forest plot of subgroup analysis of triglycerides according to outcome-based trial design.**

When stratified by follow-up duration, studies with follow-up periods of less than 1 year demonstrated a significant reduction in TAG levels (MD = −29.70 mg/dL, 95% CI: −32.64 to −26.76; P < 0.00001), with no heterogeneity (I² = 0%). Studies with follow-up durations greater than 1 year also showed a significant reduction in TAG levels (MD = −17.66 mg/dL, 95% CI: −20.33 to −14.99; P < 0.00001); however, substantial heterogeneity persisted (I² = 80%) (Figure S31). Overall, the triglyceride-lowering effect of Evolocumab remained consistent across most subgroup analyses. Heterogeneity was markedly reduced or eliminated in ACS, mechanistic, imaging, and shorter follow-up subgroups, suggesting that differences in patient populations, study design, and duration of follow-up may contribute to the variability observed in the overall TAG analysis.

**Figure S31: Forest plot of subgroup analysis of triglycerides according to follow-up duration.**

**HDL:**

For HDL cholesterol, subgroup analysis based on clinical presentation demonstrated no significant effect in ACS trials (2 studies; MD = 1.32, 95% CI: −0.91 to 3.55; P = 0.25), with no observed heterogeneity (I² = 0%). In contrast, studies enrolling patients with stable CAD/ASCVD (3 studies) showed a significant increase in HDL levels with Evolocumab (MD = 4.62, 95% CI: 2.26 to 6.99; P = 0.0001), although substantial heterogeneity remained (I² = 99%) (Figure S32).

**Figure S32: Forest plot of subgroup analysis of HDL in ACS versus stable CAD populations.**

When stratified by trial design, the single event-driven trial demonstrated a significant increase in HDL levels (MD = 7.10, 95% CI: 6.36 to 7.84; P < 0.00001), whereas the single lipid-only trial showed no significant effect (MD = 1.06, 95% CI: −2.45 to 4.57; P = 0.55). Mechanistic trials (2 studies) demonstrated a significant increase in HDL levels (MD = 4.66, 95% CI: 1.90 to 7.42; P = 0.0009) with low heterogeneity (I² = 25%). Similarly, imaging/atherosclerosis structure trials (2 studies) showed a significant increase in HDL levels (MD = 2.60, 95% CI: 2.55 to 2.65; P < 0.00001) with no heterogeneity (I² = 0%) (Figure S33).

**Figure S33: Forest plot of subgroup analysis of HDL according to outcome-based trial design.**

Subgroup analysis according to follow-up duration showed that studies with follow-up periods of less than 1 year (3 studies) did not demonstrate a statistically significant increase in HDL levels (MD = 3.15, 95% CI: −0.28 to 6.58; P = 0.07), with moderate heterogeneity (I² = 70%). Studies with follow-up durations greater than 1 year (3 studies) demonstrated a significant increase in HDL levels (MD = 3.87, 95% CI: 0.27 to 7.46; P = 0.03); however, substantial heterogeneity persisted (I² = 99%) (Figure S34). Overall, subgroup analyses reduced heterogeneity in several trial-design categories, particularly mechanistic and imaging studies, suggesting that differences in study design may partially explain the variability observed in the overall HDL analysis.

**Figure S34: Forest plot of subgroup analysis of HDL according to follow-up duration.**

**Lipoprotein (a):**

For lipoprotein(a) [Lp(a)], subgroup analysis based on clinical presentation demonstrated differing results between ACS and stable CAD/ASCVD populations. In ACS trials (2 studies), Evolocumab significantly reduced Lp(a) levels (MD = −12.45 mg/dL, 95% CI: −23.61 to −1.29; P = 0.03), although substantial heterogeneity remained (I² = 70%). In contrast, studies involving patients with stable CAD/ASCVD (3 studies) did not demonstrate a statistically significant reduction in Lp(a) levels (MD = −9.51 mg/dL, 95% CI: −25.34 to 6.32; P = 0.24), with considerable heterogeneity persisting within the subgroup (I² = 100%) (Figure S35).

**Figure S35: Forest plot of subgroup analysis of lipoprotein (a) in ACS versus stable CAD populations.**

Subgroup analysis according to trial design revealed variable treatment effects across study categories. The single event-driven trial demonstrated the largest reduction in Lp(a) levels (MD = −27.90 mg/dL, 95% CI: −30.61 to −25.19; P < 0.00001). Significant reductions were also observed in the lipid-only trial (MD = −8.10 mg/dL, 95% CI: −11.20 to −5.00; P < 0.00001) and the mechanistic trial (MD = −5.80 mg/dL, 95% CI: −7.51 to −4.09; P < 0.00001). However, imaging/atherosclerosis structure trials (2 studies) did not demonstrate a significant reduction in Lp(a) levels (MD = −6.66 mg/dL, 95% CI: −31.02 to 17.69; P = 0.59), with substantial heterogeneity observed (I² = 94%) (Figure S36).

**Figure S36: Forest plot of subgroup analysis of lipoprotein (a) according to outcome-based trial design.**

When stratified by follow-up duration, studies with follow-up periods of less than 1 year demonstrated a significant reduction in Lp(a) levels (MD = −6.57 mg/dL, 95% CI: −8.70 to −4.44; P < 0.00001), with low-to-moderate heterogeneity (I² = 38%). In contrast, studies with follow-up durations greater than 1 year did not demonstrate a statistically significant reduction in Lp(a) levels (MD = −14.12 mg/dL, 95% CI: −40.45 to 12.22; P = 0.29), and substantial heterogeneity persisted (I² = 100%) (Figure S37).

Overall, subgroup analyses suggest that the effect of Evolocumab on Lp(a) levels may vary according to patient population, trial design, and duration of follow-up. Heterogeneity was substantially reduced in the short-term follow-up subgroup, whereas considerable heterogeneity remained among stable CAD/ASCVD populations, imaging trials, and studies with longer follow-up durations. These findings indicate that differences in study characteristics may contribute significantly to the variability observed in the overall Lp(a) analysis, and the pooled results should therefore be interpreted with caution.

**Figure S37: Forest plot of subgroup analysis of lipoprotein (a) according to follow-up duration.**

**MACE:**
For major adverse cardiovascular events (MACE), subgroup analysis was performed to explore potential sources of heterogeneity and address concerns regarding the robustness of the pooled estimates. Given the substantial heterogeneity observed in the overall analysis, prespecified subgroup analyses were conducted according to clinical presentation (ACS versus stable CAD/ASCVD), trial design (event-driven versus imaging trials), and duration of follow-up (<1 year versus ≥1 year).

Based on clinical presentation, ACS trials (1 study) showed no statistically significant reduction in MACE (RR = 0.11, 95% CI: 0.01 to 2.06; P = 0.14), although interpretation is limited due to the single-study design. In contrast, stable CAD/ASCVD trials (4 studies) demonstrated a significant reduction in MACE (RR = 0.54, 95% CI: 0.40 to 0.72; P < 0.0001), albeit with substantial heterogeneity (I² = 96%) (Figure S38).

**Figure S38: Forest plot of subgroup analysis of major adverse cardiovascular events (MACE) in ACS versus stable CAD populations.**

When stratified by trial design, imaging trials (2 studies) showed a significant reduction in MACE (RR = 0.46, 95% CI: 0.36 to 0.59; P < 0.00001) with no heterogeneity (I² = 0%). Event-driven trials (3 studies) also demonstrated a significant reduction in MACE (RR = 0.58, 95% CI: 0.42 to 0.78; P = 0.0004), although considerable heterogeneity was observed (I² = 97%) (Figure S39).

**Figure S39: Forest plot of subgroup analysis of MACE according to outcome-based trial design.**

Subgroup analysis according to follow-up duration showed that studies with follow-up of less than 1 year (1 study) demonstrated a marked reduction in MACE (RR = 0.16, 95% CI: 0.10 to 0.24; P < 0.00001). In contrast, studies with follow-up durations of ≥1 year (4 studies) also showed a significant reduction in MACE (RR = 0.73, 95% CI: 0.61 to 0.87; P = 0.0006), although substantial heterogeneity persisted (I² = 88%) (Figure S40).

**Figure S40: Forest plot of subgroup analysis of MACE according to follow-up duration.**

Overall, subgroup analyses suggest that Evolocumab is associated with a reduction in MACE across most study categories; however, the magnitude of effect varies depending on trial design, clinical population, and follow-up duration. Notably, heterogeneity was minimized in imaging trials, whereas considerable inconsistency persisted in stable CAD/ASCVD and longer follow-up studies, indicating that study-level differences may partly explain the variability in the pooled estimate. These findings should therefore be interpreted cautiously in light of residual heterogeneity and limited data within certain subgroups.

**Drug Discontinuation:**

For treatment discontinuation, subgroup analysis was conducted to assess whether Evolocumab use was associated with differences in drug discontinuation rates across clinical settings and study designs. Given the limited number of studies and observed heterogeneity in certain subgroups, stratified analyses were performed according to clinical presentation (ACS versus stable CAD/ASCVD) and trial design (event-driven versus imaging trials).

Based on clinical presentation, ACS trials (1 study) showed no statistically significant difference in treatment discontinuation rates (RR = 0.41, 95% CI: 0.13 to 1.24; P = 0.11). Similarly, stable CAD/ASCVD trials (2 studies) demonstrated no significant difference in discontinuation rates between Evolocumab and control groups (RR = 0.98, 95% CI: 0.78 to 1.24; P = 0.90); however, moderate-to-substantial heterogeneity was observed within this subgroup (I² = 78%) (Figure S41).

**Figure S41: Forest plot of subgroup analysis of treatment discontinuation in ACS versus stable CAD populations.**

When stratified by trial design, event-driven trials (2 studies) showed no significant difference in treatment discontinuation rates (RR = 0.98, 95% CI: 0.78 to 1.24; P = 0.90), with substantial heterogeneity (I² = 78%). Imaging trials (1 study) also demonstrated no significant difference in discontinuation rates (RR = 0.41, 95% CI: 0.13 to 1.24; P = 0.11) (Figure S42).

**Figure S42: Forest plot of subgroup analysis of treatment discontinuation according to outcome-based trial design.**

Overall, subgroup analyses indicate that Evolocumab therapy is not associated with an increased risk of treatment discontinuation across studied populations and trial designs. However, the presence of heterogeneity in stable CAD/ASCVD and event-driven subgroups, along with the limited number of included studies, suggests that these findings should be interpreted with caution.

**Injection Site Reaction:**

For injection-site reactions, subgroup analysis was performed to evaluate whether Evolocumab use was associated with differences in adverse local reactions across clinical settings, trial designs, and follow-up duration. Given the relatively small number of included studies and variability in effect estimates, analyses were stratified according to clinical presentation (ACS versus stable CAD/ASCVD), trial design (event-driven versus imaging trials), and duration of follow-up.

Based on clinical presentation, the ACS subgroup (1 study) showed no statistically significant difference in injection-site reactions (RR = 0.34, 95% CI: 0.01 to 8.16; P = 0.50), although the estimate was highly imprecise due to the limited sample size. In contrast, stable CAD/ASCVD trials (4 studies) demonstrated a non-significant trend toward increased injection-site reactions with Evolocumab (RR = 1.65, 95% CI: 0.91 to 2.97; P = 0.10), with substantial heterogeneity observed (I² = 73%) (Figure S43).

**Figure S43: Forest plot of subgroup analysis of injection-site reactions in ACS versus stable CAD populations.**

When stratified by trial design, event-driven trials (3 studies) also showed a non-significant increase in injection-site reactions (RR = 1.59, 95% CI: 0.86 to 2.94; P = 0.14), accompanied by considerable heterogeneity (I² = 81%). In contrast, imaging trials (1 study) demonstrated no significant difference in injection-site reactions between groups (RR = 1.36, 95% CI: 0.10 to 19.06; P = 0.82), with low heterogeneity (I² = 31%) (Figure S44).

**Figure S44: Forest plot of subgroup analysis of injection-site reactions according to outcome-based trial design.**

Subgroup analysis based on follow-up duration showed a marked difference in effect estimates. The subgroup with follow-up duration of less than 1 year (1 study) reported a statistically significant increase in injection-site reactions (RR = 129.63, 95% CI: 8.07 to 2082.13; P = 0.0006); however, this finding is based on a single study with extremely wide confidence intervals, limiting interpretability. In contrast, studies with follow-up duration greater than 1 year (4 studies) demonstrated a small but statistically significant increase in injection-site reactions with Evolocumab (RR = 1.33, 95% CI: 1.14 to 1.55; P = 0.0004), with no observed heterogeneity (I² = 0%) (Figure S45).

**Figure S45: Forest plot of subgroup analysis of injection-site reactions according to follow-up duration.**

Overall, subgroup analyses indicate that while no consistent statistically significant difference is observed across most clinical and trial-level strata, longer follow-up duration may be associated with a modest increase in injection-site reactions. However, the presence of substantial heterogeneity in several subgroups and the imprecision of short-term estimates necessitate cautious interpretation of these findings, particularly given the limited number of studies within certain strata.
